# Comparative transcriptome analysis suggests convergent evolution of desiccation tolerance in *Selaginella* species

**DOI:** 10.1186/s12870-020-02638-3

**Published:** 2020-10-12

**Authors:** Gerardo Alejo-Jacuinde, Sandra Isabel González-Morales, Araceli Oropeza-Aburto, June Simpson, Luis Herrera-Estrella

**Affiliations:** 1 National Laboratory of Genomics for Biodiversity (Langebio), Unit of Advanced Genomics, CINVESTAV, 36824 Irapuato, Guanajuato Mexico; 2 Department of Genetic Engineering, CINVESTAV, 36824 Irapuato, Guanajuato Mexico; 3 StelaGenomics México, S de RL de CV, 36821 Irapuato, Guanajuato Mexico; 4 Institute of Genomics for Crop Abiotic Stress Tolerance, Texas Tech University, Lubbock, TX 79409 USA

**Keywords:** *Selaginella*, Desiccation tolerance, Convergent evolution

## Abstract

**Background:**

Desiccation tolerant *Selaginella* species evolved to survive extreme environmental conditions. Studies to determine the mechanisms involved in the acquisition of desiccation tolerance (DT) have focused on only a few *Selaginella* species. Due to the large diversity in morphology and the wide range of responses to desiccation within the genus, the understanding of the molecular basis of DT in *Selaginella* species is still limited.

**Results:**

Here we present a reference transcriptome for the desiccation tolerant species *S. sellowii* and the desiccation sensitive species *S. denticulata*. The analysis also included transcriptome data for the well-studied *S. lepidophylla* (desiccation tolerant), in order to identify DT mechanisms that are independent of morphological adaptations. We used a comparative approach to discriminate between DT responses and the common water loss response in *Selaginella* species. Predicted proteomes show strong homology, but most of the desiccation responsive genes differ between species. Despite such differences, functional analysis revealed that tolerant species with different morphologies employ similar mechanisms to survive desiccation. Significant functions involved in DT and shared by both tolerant species included induction of antioxidant systems, amino acid and secondary metabolism, whereas species-specific responses included cell wall modification and carbohydrate metabolism.

**Conclusions:**

Reference transcriptomes generated in this work represent a valuable resource to study *Selaginella* biology and plant evolution in relation to DT. Our results provide evidence of convergent evolution of *S. sellowii* and *S. lepidophylla* due to the different gene sets that underwent selection to acquire DT

## Background

**Fig. 1:**
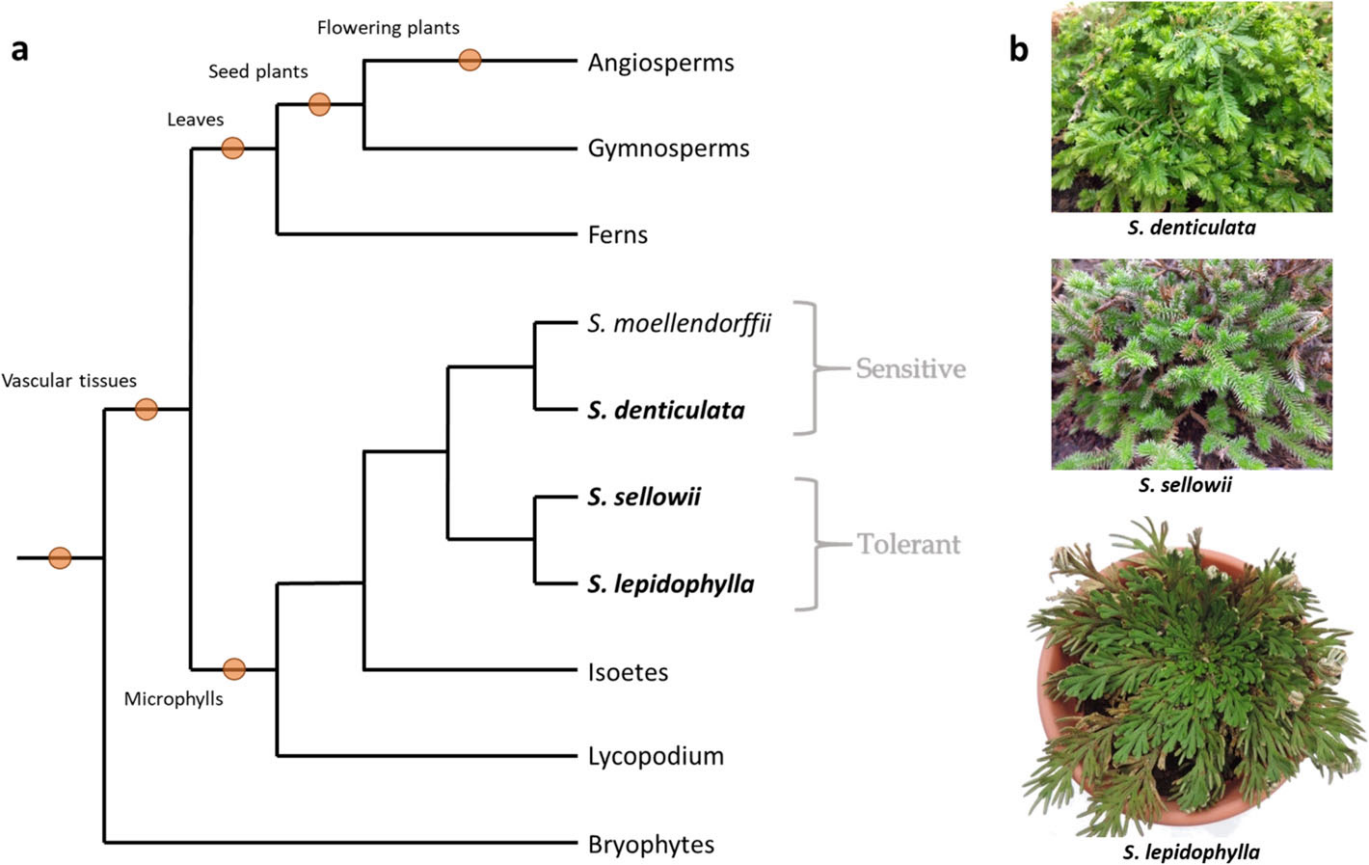
Cladogram of major groups of land plants showing the position of the *Selaginella* species used in this study. **a** The orange circle indicates evolutionary innovations in each group of plants. *Selaginella* species analyzed in this work are shown in bold and dessication response in grey. **b** Examples of the morphology of each of the species analyzed. Features and positions of major groups of land plants adapted from [2] and consensus phylogenetic relationships between *Selaginella* species from [1, 3–6]

The origin of the *Selaginella* genus has been estimated at around 383 million years ago [1]. This group of plants represents one of the oldest lineages of vascular plants (Fig. 1)⁠ and includes over 700 species [2, 7, 8]⁠. The *Selaginella* genus occupies a broad diversity of habitats, mainly in humid environments, however some species are adapted to extremely arid conditions [1, 3]⁠. Some of these latter species have evolved desiccation tolerance (DT), a particular trait that allows them to withstand very long periods in the desiccated state. Tolerance to desiccation, considered as the ability to recover from the almost complete loss of protoplasmic water, is widespread in reproductive structures (such as seeds and pollen) but uncommon in the vegetative tissues of tracheophytes [9]. A substantial number of *Selaginella* species occupy extreme dry habitats and at least 15 members have been classified as desiccation tolerant [10–12]⁠. Mechanisms of DT in these species include accumulation of polyols such as sorbitol and xylitol that act as osmoprotectants by stabilizing protein structure, activation of flavonoid and glutathione metabolism to prevent oxidative stress [13], increased accumulation of proline and a burst of antioxidative enzymes [11]. Tolerant *Selaginella* species have been classified as homoiochlorophyllous because they retain chlorophyll and thylakoid membranes during desiccation [14]. Morphological mechanisms such as stem curling and leaf folding also contribute to DT in these species by limiting light harvesting and the consequent formation of reactive oxygen species (ROS) due to the retention of photosynthetic apparatus [15–17]⁠. The recent genome sequencing of the tolerant species *Selaginella lepidophylla* [18]⁠ and *S. tamariscina* [19]⁠ has also revealed new insights into the molecular basis of desiccation. The *S. lepidophylla* genome (109 Mb) has tandem gene duplications associated with its adaptation to extreme water loss, specifically gene family expansions in Early Light-Induced Proteins (ELIPs) and Late Embryogenesis Abundant (LEA) proteins [18, 20]⁠. The proposed function of ELIPs is to prevent oxidative damage by binding to photosynthetic pigments. Analysis comparing all available sequenced desiccation tolerant genomes suggests that convergent evolution occurred in the expansion of ELIP genes independently [21]⁠, supporting the hypothesis of convergent evolution of DT. Furthermore, in the *S. tamariscina* genome (301 Mb) a significant expansion of oleosin genes that have a putative function in energy storage and membrane repair was also identified [19]⁠*.* Additionally, this species also presents modified mechanisms of generation and scavenging of ROS compared to its sensitive relative *S. moellendorffii*. These findings show that even species from the same genus may have evolved common as well as specific strategies to acquire DT.

*Selaginella* has a large diversity of ecological niches, growth forms and morphologies [22]⁠. Morphologically, there are two major groups within the genus of which the largest is represented by anisophyllous species (four ranked microphylls; two dorsal and two ventral rows) found mostly in wet tropical forests. The other group comprises isophyllous species (indistinct rows, helically arranged microphylls), commonly found in arid regions of Mexico and the western United States [4, 23]⁠. Most research on DT in *Selaginella* to date has been carried out in anisophyllous species. Since *Selaginella* species classified as tolerant to desiccation can show widely distinct morphologies it is of fundamental interest to determine if both morphological types have shared and/or specific mechanisms that mediate their DT capacity.

Here we report comparative RNA-Seq analysis at different time points of the dessication process of the isophyllous plant species *S. sellowii* and the anisophyllous species *S. denticulata.* The study of *S. sellowii* has previously focused on pharmacological applications due to its potential use in the treatment of cutaneous leishmaniasis [24], a major health problem in Central and South America. *S. sellowii* was first reported as desiccation tolerant by Gaff in 1987 [25], but its molecular mechanisms of DT has not been characterized. *S. denticulata* is also a species of interest in which several biflavonoids with potential pharmacological properties have been identified [26].

Previous studies using comparative approaches to dissect DT traits have included physiological [27, 28]⁠, metabolic [13, 29]⁠, gene expression [30, 31]⁠, and genome analysis [32, 33]. The present study compares the transcriptional responses during dehydration and rehydration of two tolerant species with highly different morphologies (*S. sellowii* and *S. lepidophylla*) with the response in a sensitive species (*S. denticulata*). Our findings show that closely related species could have evolved DT by adaptation based on different genes/proteins that led to the implementation of very similar tolerance mechanisms.

## Results

### Characterization of DT in *Selaginella sellowii*

**Fig. 2:**
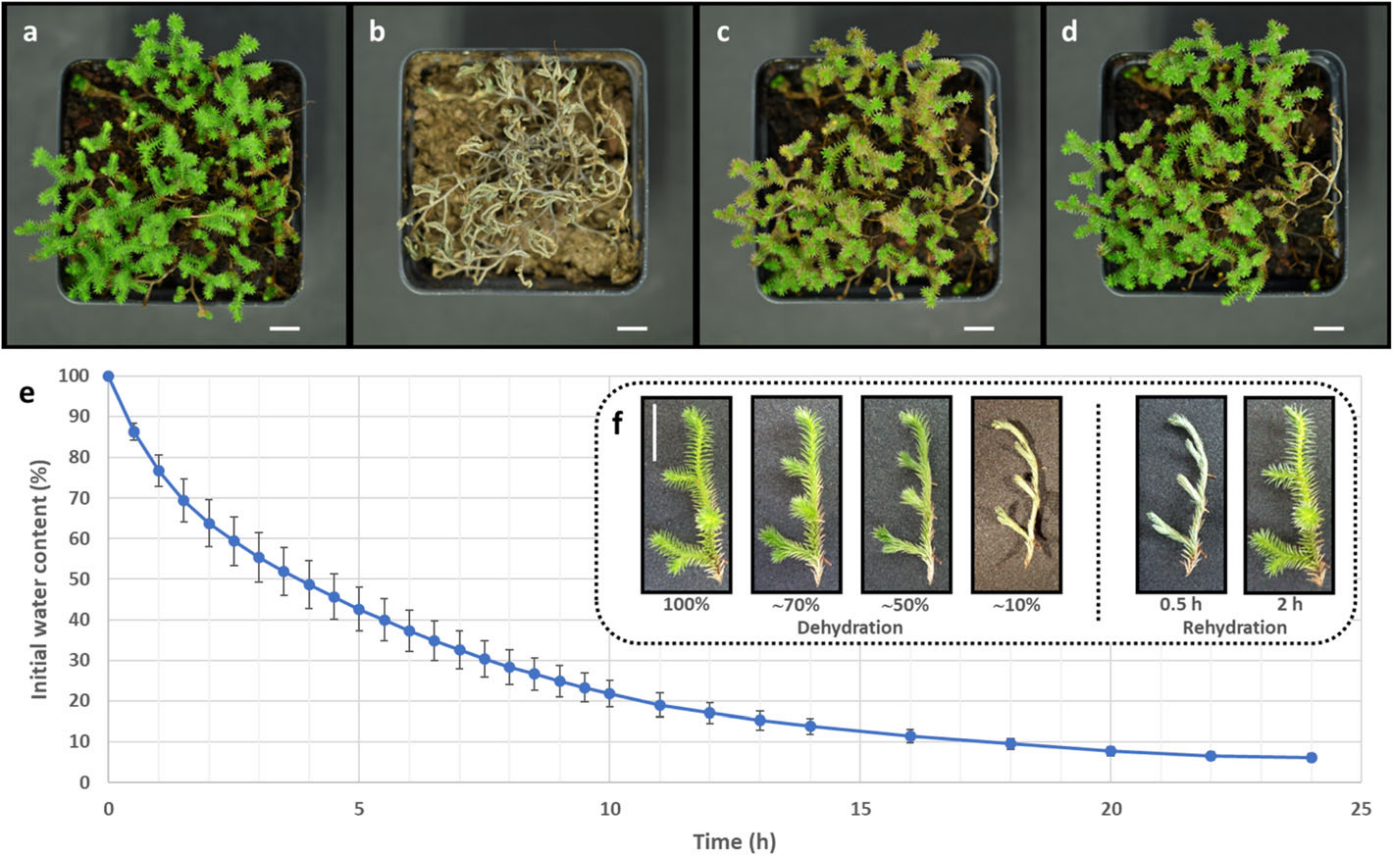
Desiccation tolerance and drying rate of *S. sellowii* explants.​ Greenhouse desiccation experiments, the same individual pot is shown in **a** hydrated or well watered conditions, **b** desiccated state (1 month withholding water), **c** 48 h after rehydration, **d** 1 week after rehydration. **e** Dehydration of explants expressed as the percentage loss of initial water content, points represent mean values of 4 replicates ± SD. **f** Changes in morphology during water loss and rehydration, scale bar 1 cm

To identify shared or specific functions relevant to the acquisition of DT in *Selaginella* species with different morphologies, and to discriminate between the shared and conserved responses to water loss from the response to desiccation, we compared RNA-Seq data from two *Selaginella* species with similar morphologies that differ in DT properties with a tolerant species with a distinct morphology. The morphology and phylogenetic positions of the *Selaginella* species chosen for this analysis are shown in Fig. 1. The cladogram is a representation of previous phylogenetic studies that have shown concordance in the relationships between *S. sellowii*, *S. lepidophylla* and *S. denticulata* using different molecular markers [1, 3–6]. Although *S. sellowii* has previously been reported as desiccation tolerant, we decided to confirm this evaluation with our samples. Therefore *S. sellowii* plants were subjected to a dehydration and rehydration cycles under greenhouse conditions in order to determine their DT capacity. The phenotypes of representative specimens of *S. sellowii* during this process are shown in Fig. 2. Individual pots were exposed to dehydration by withholding water for a month. Most water loss occurred in the first 12 days, during which the tissue reached a desiccated state (Fig. 2b). Over that period, the greenhouse registered a mean relative humidity of 40.35 ± 7.15%, an adequate environment to determine DT [34]. After a long period in the dry state rehydrated tissue of *S. sellowii* presented a slightly brown pigmentation (Fig. 2c) but after 1 week of rehydration recovered a similar color to that observed before water was withheld (Fig. 2d). To test whether detached *S. sellowii* branchlets (referred to as explants) are also tolerant and maintain viability upon desiccation, this tissue was also subjected to repeated cycles of dehydration-rehydration. The *S. sellowii* explants were also tolerant with no apparent damage after recurrent desiccation whereas explants of the sensitive species *S. denticulata* clearly lost viability (Additional file 1: Figure S1a).

To more carefully examine the DT response in *S. sellowii*, explants were dehydrated under similar conditions to the greenhouse experiments (46.96 ± 4.19% relative humidity). The drying rate of *S. sellowii* explants was determined by the percentage loss of initial water content (Fig. 2e). Under the experimental conditions it was found that *S. sellowii* explants lost 30% of initial water content 90 min after initiation of the dehydration treatment, 50% after 4 h, 70% after 7.5 h and 90% after 17 h (Fig. 2e). Microphyll folding in *S. sellowii* explants, thought to protect the tissue from oxidative damage during desiccation, was evident below 50% of water content (Fig. 2f). During rehydration *S. sellowii* explants rapidly recovered and microphylls were fully opened at 2 h after rehydration (Fig. 2f). The detached explants assay was also carried out for *S. lepidophylla* and *S. denticulata*. Similar results in drying rate were obtained for the tolerant species *S. lepidophylla*, although morphological changes during water loss are predominantly stem curling rather than microphyll folding. Another difference of *S. lepidophylla* in comparison to *S. sellowii* explants is that the fully hydrated morphology was not completely recovered at 2 h after rehydration (Additional file 1: Figure S1b). The desiccation sensitive *S. denticulata* showed a more rapid water loss (reaching 10% of its initial water content after 9 h of treatment) but no microphyll folding or stem curling was observed (Additional file 1: Figure S1c). Furthermore, *S. denticulata* explants lost turgidity when water was withheld and there was evident tissue oxidation during rehydration.

### Development of reference transcriptome assemblies for *S. sellowii*, *S. lepidophylla* and *S. denticulata*

To obtain a global overview of changes in gene expression patterns during the DT process, we extracted RNA from fully hydrated, partially dehydrated (70 and 50% of initial water content), and fully dehydrated (10% of initial water content) explants of *S. sellowii*, *S. lepidophylla* and *S. denticulata* (samples were collected according to their respective drying curves; Fig. 2 and Additional file 1: Figure S1). Explants from *S. sellowii* and *S. lepidophylla* were further desiccated to less than 10% initial water content, and rehydrated for 2 and 6 h to analyze transcriptional responses during recovery. RNA-Seq analysis of *S. denticulata* was only carried out for the dehydration process since tissue damage and loss of RNA integrity was reproducibly observed following rehydration (Additional file 2: Figure S2).

Sequencing was performed using the NextSeq Illumina platform with a paired-end (2 × 75 bp) read format. Raw sequencing reads were filtered to remove low-quality and adapter sequences to obtain a total output of more than 100,000,000 reads for each species (Table 1). Reads from the three *Selaginella* species were assembled separately using Trinity [35]⁠ and SOAPdenovo-Trans [36]⁠ algorithms. Evaluation of assembly quality included a combination of metrics such as N50 length, gene prediction, completeness, and proportion of reads mapping to the assembly (Table 1). Trinity de novo assemblies were selected as they showed a better performance in each metric.

**Table 1:** Summary of *Selaginella* assemblies

	***S. sellowii***		***S. lepidophylla***		***S. denticulata***	
Total Raw Reads	138,288,472		199,259,295		114,231,403	
Total Trimmed Reads	125,956,200		182,787,767		104,700,744	
	**Trinity**	**SOAPdenovo**	**Trinity**	**SOAPdenovo**	**Trinity**	**SOAPdenovo**
**rnaQUAST**						
Contigs	56,287	48,843	64,189	66,262	62,111	60,458
Contigs > 1000 bp	***37,086***	10,561	***31,815***	10,277	***32,281***	14,454
Longest contig (bp)	27,152	39,804	22,517	38,874	20,697	21,926
Average length (bp)	2631.69	953.46	1962.86	780.02	1650.37	798.22
N50 length	***4482***	4100	***4061***	4057	***2825***	2397
Total Assembled Bases	148,130,112	46,570,041	125,993,766	51,685,348	102,506,311	48,258,718
GC content (%)	53.22%	52.92%	50.84%	50.93%	49.74%	49.54%
Predicted genes (GeneMarkS-T)	***27,005***	8,189	***29,728***	10,146	***32,874***	14,626
**BUSCO**						
Complete	***95.10%***	70.60%	***93.10%***	69.00%	***98.30%***	88.10%
**Bowtie2**						
Overall Alignment Rate	***96.17%***	87.38%	***95.08%***	82.89%	***96.99%***	88.48%
**DETONATE**						
RSEM-EVAL score	***−6.50 × 10***^***9***^	−8.20 × 10^9^	***−10.4 × 10***^***9***^	−14.3 × 10^9^	***−5.34 × 10***^***9***^	− 6.95 × 10^9^

Quality metrics of de novo transcriptome assemblies using Trinity and SOAPdenovo-Trans assemblers. The best values of relevant metrics are indicated in bold italic. BUSCO: Benchmarking Universal Single-Copy Orthologs. DETONATE: DE novo TranscriptOme rNa-seq Assembly with or without Truth Evaluation

Benchmarking Universal Single-Copy Orthologs (BUSCO) analysis [37] indicated a high degree of coverage with presence of the 95, 93 and 98% of the eukaryotic markers in *S. sellowii*, *S. lepidophylla* and *S. denticulata* assemblies, respectively (Table 1). However, most of the BUSCO markers (single-copy orthologs) were detected as duplicated in the assemblies (up to 70% for *S. sellowii*; Table 2). To decrease redundancy and define a reference transcriptome for each species, only the longest isoform per contig was retained (for subsequent analysis referred to as transcripts). After reduction of redundancy 41.6, 50.5 and 61.2% of the total contigs for *S. sellowii*, *S. lepidophylla* and *S. denticulata*, were retained respectively. Assemblies were largely complete as more than 92% of raw reads aligned to their respective assembly (Table 2). Although this strategy leads to lower quality metrics such as N50 length, a significant improvement in the level of redundancy was obtained since the number of complete single-copy markers increased to > 75% in each species (Table 2).

**Table 2:** Completeness and redundancy of *Selaginella* assemblies

	***S. sellowii***		***S. lepidophylla***		***S. denticulata***	
	Complete	Longest isoforms	Complete	Longest isoforms	Complete	Longest isoforms
**rnaQUAST**						
Contigs	56,287	23,429	64,189	32,386	62,111	38,018
Contigs > 1000 bp	37,086	8,080	31,815	7,684	32,281	13,344
Average length (bp)	2631.69	1,309.19	1,962.86	1,036.79	1,650.37	1,094.03
N50 length	4,482	3,007	4,061	2,789	2,825	2,034
Total Assembled Bases	148,130,112	30,673,108	125,993,766	33,577,579	102,506,311	41,592,701
**BUSCO**						
Complete (single copy)	24.8%	***77.2%***	25.1%	***75.6%***	52.1%	***77.9%***
Complete (duplicated)	70.3%	7.9%	68.0%	13.2%	46.2%	17.2%
Fragmented	3.3%	12.5%	4.0%	7.9%	0.7%	4.0%
Missing	1.6%	2.4%	2.9%	3.3%	1.0%	0.9%
**Bowtie2**						
Overall Alignment Rate	96.17%	93.86%	95.08%	92.25%	96.99%	94.57%

The best values of relevant metrics are indicated in bold italic

Transcriptomes were annotated using BLASTX against SwissProt and RefSeq (plant) databases and protein models of several species including *Arabidopsis thaliana*. The percentage of annotation of each transcriptome was 55.72, 55.43 and 52.29% for *S. sellowii*, *S. lepidophylla* and *S. denticulata*, respectively. At least 42% of the transcripts in each species showed significant similarity with protein models of the previously sequenced *S. moellendorffii* [38]⁠. During dehydration (DH) and rehydration (RH) of *S. sellowii* and *S. lepidophylla* explants, a total of 4,001 and 5,098 transcripts were differentially expressed respectively of which 2,908 and 3,455 respectively were at least partially annotated. In *S. denticulata* explants we observed 5,738 differentially expressed transcripts of which 4,033 could be annotated. Additionally, size distribution of the responsive transcripts showed a strong correlation between sequence length and annotation (Additional file 3: Figure S3).

### Proteome of *Selaginella* species

**Fig. 3:**
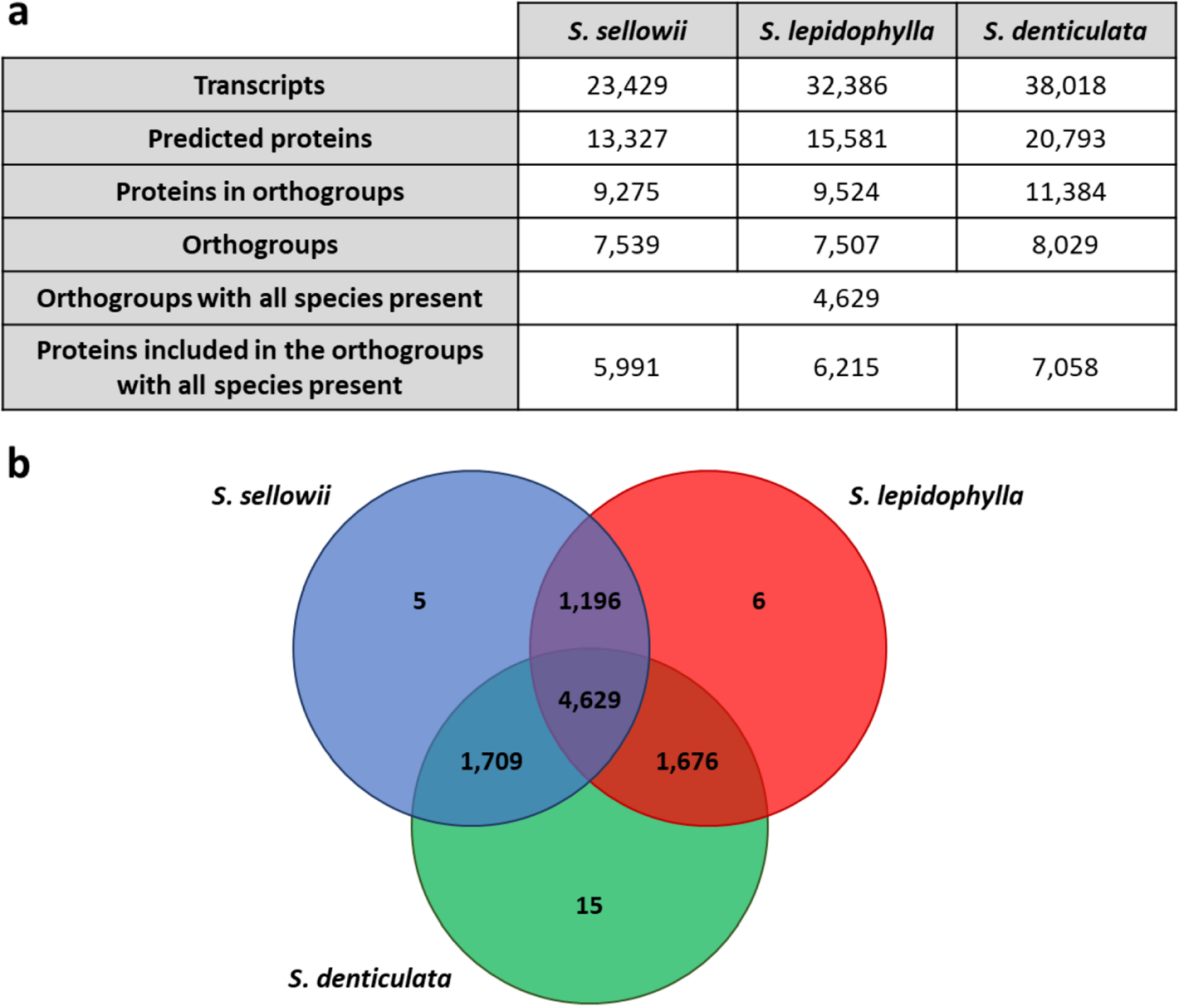
Orthogroups shared between *Selaginella* species. **a** Number of protein families identified using OrthoFinder. **b** Venn diagram illustrating shared and specific orthogroups

Protein sequences were predicted from the reference transcriptomes (longest isoforms) using TransDecoder. Inferred proteomes of *Selaginella* species showed a variable size, where the numbers of transcripts determined to have coding potential were 13,327, 15,581 and 20,793 for *S. sellowii*, *S. lepidophylla* and *S. denticulata*, respectively (Fig. 3a). Clustering using OrthoFinder [39]⁠ defined a total of 9,236 orthogroups for the predicted proteomes, showing a high similarity between all *Selaginella* species. Around half of the protein families shared homology in all three species, the remainder were largely shared in combinations of each pair of species and only a few were classified as species-specific protein families by this analysis (a total of 26 orthogroups) (Fig. 3b). Although the number of orthogroups shared between the sensitive species and each of the tolerant species was greater than that between the tolerant species, the percentage of sequence identity determined by reciprocal best hits (RBH) analysis was highest (around 71%) between the tolerant species *S. sellowii* and *S. lepidophylla* as compared to 65–66% for each in comparison to *S. denticulata* (Additional file 4: Figure S4).

### Shared and contrasting transcriptional profiles between *Selaginella* species

**Fig. 4:**
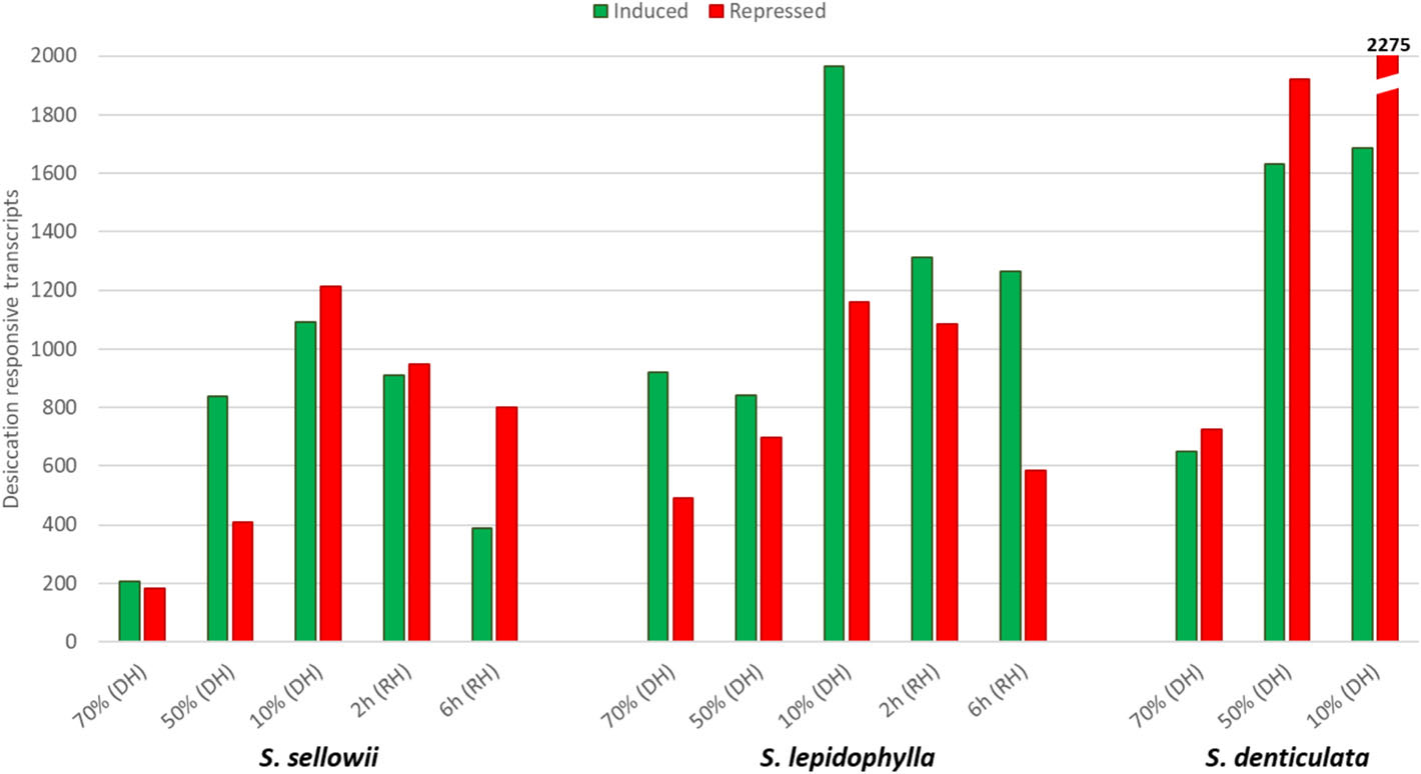
Number of desiccation responsive transcripts. Transcripts responsive to dehydration (DH) and rehydration (RH) were determined by a threshold of logFC ≥ 1 (induced, green bars) or ≤ − 1 (repressed, red bars) and FDR < 0.01 regarding the initial condition (100%)

To examine the transcriptional response to desiccation in *Selaginella*, tissue samples were harvested during several points of DH and RH. Desiccation responsive transcripts under each condition were detected by quantification of their expression relative to hydrated samples. During DH, both tolerant and sensitive species showed a similar pattern, namely an increase in the number of responsive transcripts in relation to water loss (Fig. 4). The maximum number of responsive transcripts was observed at extreme DH (10% water content) with 1,091, 1,964 and 1,686 induced, whereas 1,214, 1,160 and 2,275 repressed transcripts were identified for *S. sellowii*, *S. lepidophylla* and *S. denticulata* respectively. Tolerant species were also analyzed during RH and showed a decrease in responsive transcripts at longer times after RH (6 h), when the tissue had time to recover the initial condition (Fig. 4). At 6 h after RH the numbers of induced transcripts were 389 and 1,263, whereas repressed transcripts were 799 and 583 for *S. sellowii* and *S. lepidophylla*, respectively. *S. lepidophylla* showed a higher level of induced rather than repressed transcripts under each condition of the DT process (1.20 to 2.16-fold more induced than repressed). *S. sellowii* initially showed a similar pattern at 70 and 50% water content (1.13–2.05-fold more induced than repressed), but at extreme DH (10% water content) and RH showed a higher proportion of repressed transcripts (1.04 to 2.05 fold more repressed over induced). Expression changes in the sensitive species were mainly associated with repressed transcripts (1.12 to 1.34-fold more repressed than induced).

### Tolerant *Selaginella* species show significant differences in gene expression patterns in response to desiccation

**Fig. 5:**
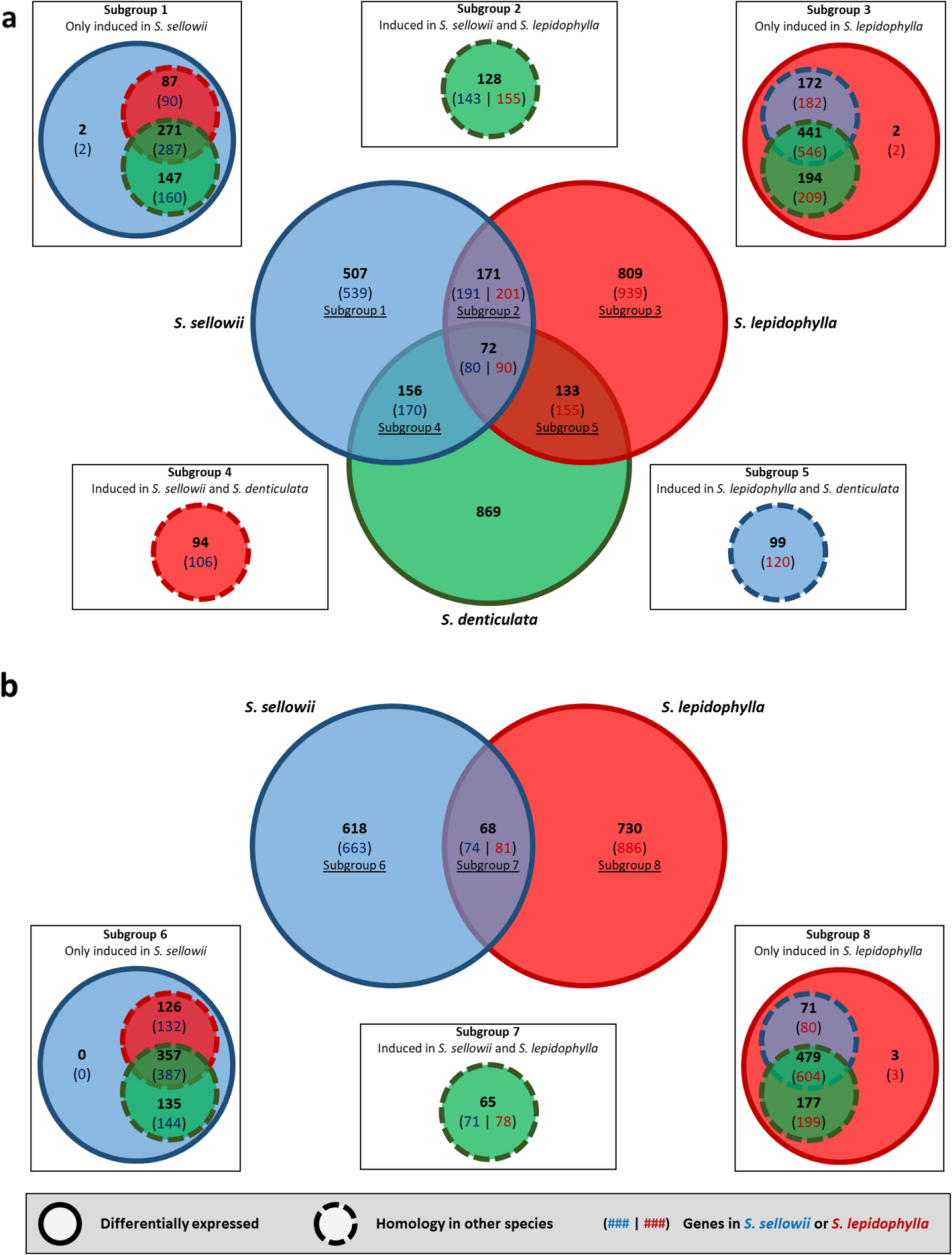
Comparison of desiccation responsive genes during dehydration and rehydration. **a** Orthogroups induced during the dehydration process (70, 50 and 10% water content) in *S. sellowii* (blue), *S. lepidophylla* (red), and *S. denticulata* (green). **b** Orthogroups induced during the rehydration process (2 and 6 h after watering) of tolerant species only. Orthogroup analysis of each intersection are indicated as subgroups where which dashed circles indicate homology without differential expression. Number of genes corresponding to *S. sellowii* (blue) and *S. lepidophylla* (red) are shown in brackets

All dehydration responsive transcripts induced at 70, 50 and 10% of water content were analyzed together for a better comparison of the DH process between tolerant and sensitive species. Using orthogroups to compare the desiccation responses between species, we found divergence in the genes activated by DH. Despite the fact that *Selaginella* proteomes show very high homology between them (Fig. 3), common responses to DH were represented by only 7.94, 6.07 and 5.85% of the total number of induced orthogroups in *S. sellowii*, *S. lepidophylla* and *S. denticulata*, respectively and a total of 72 orthogroups represented the common DH response (Fig. 5a). As expected, a larger portion of common orthogroups were shared by species with DT ability than between the tolerant and sensitive species; the total shared response to DH between tolerant species was 26.82 and 20.5% for *S. sellowii* and *S. lepidophylla*, respectively. A detailed analysis of the differentially induced orthogroups (subgroups 1 and 3 from Fig. 5a) revealed that these were composed of 539 and 939 genes with at least one homolog in the other species (except for 4 species-specific genes) but only responsive to DH in *S. sellowii* and *S. lepidophylla* respectively (Fig. 5a).

Similarly, orthogroups of the RH timepoints (2 and 6 h after watering) of the tolerant species were analyzed and showed a shared response of only 9.91 and 8.52% for *S. sellowii* and *S. lepidophylla*, respectively (Fig. 5b). The number of genes identified as specifically induced by RH (subgroups 6 and 8 from Fig. 5b) was 663 for *S. sellowii* and 886 for *S. lepidophylla*. All responsive genes in *S. sellowii* have homology in the other species and only 3 were found to be species-specific for *S. lepidophylla*. These differences suggest that during the evolution of *Selaginella* differential expression of distinct gene sets was selected in order to acquire DT.

Orthogroups could consist of a variable number of homologous sequences per species, which complicate data validation of differential expression between homologs within specific orthogroups. Therefore, single copy orthogroups (one homolog per species) that were differentially induced in tolerant species were selected for validation by quantitative real-time PCR (qRT-PCR). Genes were selected based on annotation results and previous reports of a putative role in DT. Selected orthogroups were OG5138, OG4174 and OG2082, as well as a gene annotated as ubiquitin protein ligase (OG3782) that showed no differential expression between the DH or RH treatment in the different species and was used as a reference gene. Expression levels at extreme dehydration (10% water content) and early rehydration (2 h) were evaluated with respect to hydrated conditions. Transcriptome data showed upregulation of the OG5138 orthogroup during DH and RH specifically in tolerant species; this expression pattern was confirmed by qRT-PCR (Additional file 5: Figure S5). OG5138 is orthologue to an *Arabidopsis thaliana* PLATZ transcription factor (AT1G21000), that was reported as part of the regulatory network controlling seed desiccation tolerance in *Arabidopsis* [40]. OG2082 enconde a LEA protein (AT2G44060) that showed a significant upregulation only in *S. lepidophylla* (Additional file 5: Figure S5). OG4174 genes were annotated as a pectin methylesterase inhibitor (AT5G09760) with a putative role in cell wall modification and qRT-PCR results showed upregulation during both DH and RH in *S. sellowii* but only during DH in *S. lepidophylla* (Additional file 5: Figure S5). qRT-PCR results were in general consistent with the expression levels obtained by RNA-Seq data for the different species tested.

### Common and specific mechanisms involved in the acquisition of DT in *Selaginella*

**Fig. 6:**
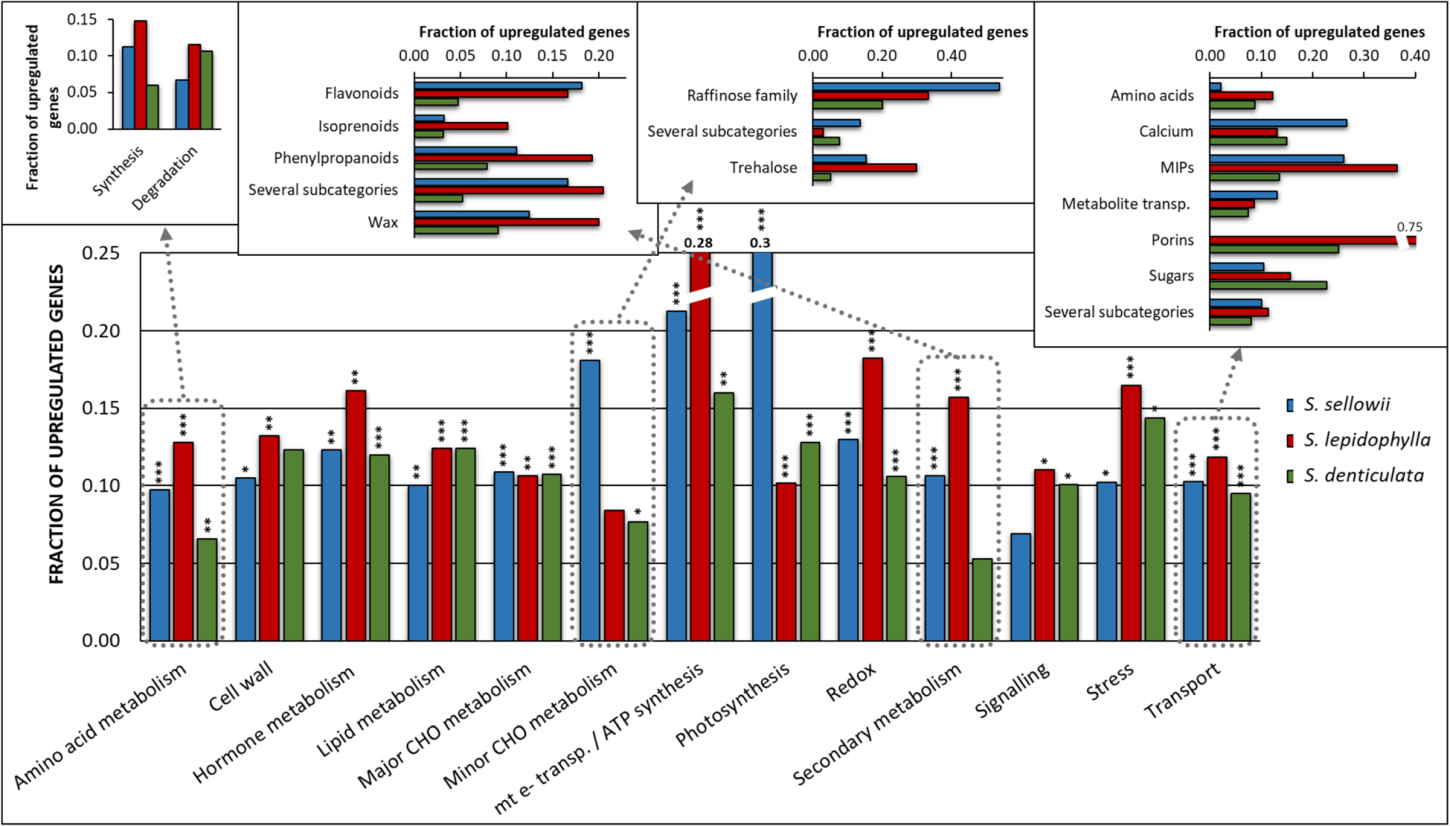
Functional analysis of genes induced during dehydration in relation to the whole transcriptome. Each MapMan category is represented as the fraction of the number of induced genes over the total number of the same category in the reference transcriptome assembly of *S. sellowii* (blue), *S. lepidophylla* (red) and *S. denticulata* (green). Some outstanding categories are divided into subcategories. Significance categories per species: *P-value< 0.05, **P-value< 0.01, ***P-value< 0.001

To determine the putative mechanisms involved in *Selaginella* DT in each species, transcripts induced in response to DH and RH were assigned functions based on MapMan terms. To compare between species each category was expressed as the fraction of induced genes of such category relative to its presence in the reference transcriptomes. For the DH stage, categories enriched in the sensitive plant *S. denticulata* were used as a reference to determine which categories could be involved in determining tolerance. The most relevant induced categories common to tolerant species with a significant difference in the sensitive species were: amino acid, secondary metabolism, redox and transport (Fig. 6). The amino acid category of the tolerant species contained 19 and 36 genes involved in synthesis in comparison to 5 and 8 involved in degradation for *S. sellowii* and *S. lepidophylla* respectively. In contrast, the sensitive species *S. denticulata* showed 10 genes for amino acid synthesis and 11 for degradation (Additional file 9: Table S1). The fraction of differentially upregulated genes for antioxidant compounds, such as glutathione, ascorbate, peroxiredoxin and thioredoxin, was 1.31 to 2.28 fold higher in tolerant species compared to sensitive species (Additional file 6: Figure S6g). Despite the fact that induction of antioxidant enzymes has been reported in tolerant species, only *S. lepidophylla* exhibited a significant enrichment in this category, such as dismutases and catalases that were enriched 2.7 times in comparison to *S. denticulata* (Additional file 6: Figure S6g). The secondary metabolism category was significantly enriched in tolerant species including genes implicated in the synthesis of several compounds known to be involved in water stress responses such as flavonoids, phenylpropanoids and wax (Fig. 6; Additional file 7: Figure S7e). The phenylpropanoid subcategory included lignin biosynthesis and the fraction of induced genes was 1.68 and 2.53 fold higher in *S. sellowii* and *S. lepidophylla* respectively, than in *S. denticulata*. Transport related functions were highly variable between tolerant species and some significant subcategories were calcium transport for *S. sellowii* and porins for *S. lepidophylla* throughout the desiccation process (Fig. 6; Additional file 7: Figure S7c). Both tolerant species mainly coincided in expression patterns of major intrinsic proteins (MIPs) with 26 and 36% induction of all classified MIPs for *S. sellowii* and *S. lepidophylla* respectively (Fig. 6). In *S. denticulata*, expression of MIP genes was also induced but only to around 14% of these. The MIPs significantly enriched during dehydration in *S. lepidophylla* were mainly classified as PIP followed by TIP type (9 and 5 transcripts), a similar result but in lower proportions was found in *S. sellowii* (3 and 2 transcripts; Additional file 10: Table S2). Significantly enriched categories during RH were similar to those determined for DH in both tolerant species with the exception of nitrogen metabolism and some MIPs genes that remained activated during RH in *S. lepidophylla* (Additional file 7: Figure S7).

Functional analysis suggest that the cell wall category was significantly enriched in a species-specific manner for *S. lepidophylla* because it included the expression of 44 genes during DH and 39 during RH, whereas *S. sellowii* only showed 21 and 11 genes during DH and RH, respectively (Additional file 11: Table S3). The processes that belong to this category suggest a remodeling of the cell wall during desiccation and included genes involved in cell wall modification (e.g. XTH, expansins) and degradation, cell wall specific proteins (e.g. AGPs) and cell wall precursors (Additional file 6: Figure S6a; Additional file 7: Figure S7a). Additionally, a higher number of pectin esterases were induced in tolerant *Selaginella* species in comparison to the sensitive species (Additional file 6: Figure S6a). These results suggest that as previously reported cell wall modification plays an important role in desiccation tolerance plants including *Selaginella* species [41]. Minor carbohydrate metabolism showed a significant difference between tolerant species during DH, where *S. sellowii* showed 7 differentially induced transcripts classified as raffinose family genes (Fig. 6; Additional file 9: Table S1) an oligosaccharide that has not been detected in *Selaginella* species. Moreover, trehalose metabolism genes (specifically TPP) were only induced during dehydration in tolerant species (Additional file 9: Table S1).

*S. sellowii* showed a significant enrichment of photosynthesis related terms during DH (Fig. 6). A detailed analysis of this function determined a higher number of induced rather than repressed genes mainly in Calvin cycle and Light-reaction subcategories (19/1 and 82/5 induced over repressed respectively) whereas *S. lepidophylla* presented a slightly higher number of induced rather than repressed genes in each subcategory (14/6 and 26/25), similar numbers of induced and repressed genes were detected in *S. denticulata* (15/14 and 44/44) for each subcategory respectively (Additional file 8: Figure S8a). The induction of photosynthesis related genes in *S. sellowii* during DH could indicate a role in the rapid recovery of photosynthetic activity at the RH stage. This was supported by measurements of photosynthetic rates that determined a faster recovery of photosynthesis in *S. sellowii* in comparison to *S. lepidophylla* (Additional file 8: Figure S8b). Recovery was also evaluated by maximum quantum efficiency of PSII showing higher values in *S. sellowii* at early RH times (0.33 and 1 h) even when no positive photosynthesis values were registered (Additional file 8: Figure S8c).

ELIPs are a key component for the protection of photosynthetic machinery during desiccation. Transcriptome data for *S. lepidophylla* showed significant induction of 5 ELIP genes across all DH treatments (70, 50 and 10% water content), whereas *S. sellowii* showed induction of 4 ELIP genes at 50% that was maintained at 10% water content. In both tolerant species the ELIP genes were highly expressed at early RH (2 h) and switched off at 6 h after RH. ELIP genes were also induced in the sensitive species *S. denticulata* but only at 50% of water content.

## Discussion

Desiccation studies in the *Selaginella* genus have gained relevance in recent years due to its phylogenetic position as one of the earliest diverging genera of vascular plants. The identification of desiccation tolerant species with widely different morphologies in this basal tracheophyte lineage allowed us to shed light on the relationship between morphology and DT mechanisms. Therefore, the comparative RNA-Seq analysis in this study included tolerant species with contrasting morphologies in order to determine mechanisms involved in *Selaginella* DT that are either independent or dependent on morphological adaptations.

Although *S. lepidophylla* and *S. tamariscina* have been studied extensively in relation to DT, less information is available for other tolerant *Selaginella* species with different morphologies such as *S. sellowii* (isophyllous), whose DT capacity was corroborated in this study at the plant and explant level. Since using intact plants could have the disadvantage of uncovering differential expression patterns due to developmental regulation or long-distance signaling during desiccation [42], we designed a system based on the use of explants to study the transcriptional effects of DH and RH treatments. The explant method proved to be a simple and reproducible technique which allows the relatively rapid analysis of replicated *Selaginella* samples.

The rosette morphology of *S. lepidophylla* produces a compact sphere with very precise stem packing when drying. Some of the factors associated with such mechanical capacity are cell density (asymmetric between abaxial and adaxial sides) and differences in the chemical composition between inner and outer stems that modify their capacity to lose water [16, 43]. Since in *S. lepidophylla* exposed to dehydration the outer stems dry faster than inner stems producing a different water status between branches of the same plant, the use of explants represents a simpler and more homogeneous approach to analyze the molecular and physiological responses of *Selaginella* species at specific water contents.

To obtain adequate and accurate references for transcriptome data of subsequent comparative analysis, different assembly algorithms were tested. De novo transcriptome analysis software such as Trinity and SOAPdenovo-Trans, have been classified as some of the most accurate assemblers [44] and although the two pipelines produced similar numbers of contigs, relevant quality metrics (N50 length, number of predicted genes, and proportion of mapped reads) indicated a better performance for Trinity for all *Selaginella* species. The RSEM-EVAL score, a complementary analysis to evaluate assembly accuracy [45], also evaluated Trinity assemblies with higher scores. Analysis based on BUSCO gene sets, as an indicator of assembly completeness [37],⁠ also indicated that Trinity assemblies were largely complete. Since the Trinity algorithm generates alternatively spliced isoforms of the RNAs produced by a single gene, to eliminate redundancy for further comparative analysis between species, each assembly was filtered for the longest isoforms per contig. BUSCO analysis showed that using the longest isoform of each transcript diminished redundancy without a significant universal loss of gene coverage.

Predicted proteomes were subjected to OrthoFinder analysis to define orthogroups or protein families within the *Selaginella* species. Results of this analysis showed a significant number of common protein families (50% of the orthogroups were common to all species) and only a few were classified as species-specific (< 0.01% of the total orthogroups). An unexpected finding was that a larger number of orthogroups was shared between *S. denticulata* and the tolerant species than between the tolerant species, this may be because the *S. denticulata* transcriptome was the most complete according to BUSCO results, whereas the protein sequences of tolerant species showed greater mean sequence identity. The latter observation agrees with previous phylogenetic analyses that have shown that *S. sellowii* and *S. lepidophylla* species are phylogenetically more closely related [3–6]. Although there are obvious morphological differences between *S. lepidophylla* and *S. sellowii*, historical biogeographic studies have shown that both shared a common ancestor in the late Permian with an early adaptation to arid regions [1].

*S. lepidophylla* had a higher number of induced rather than repressed transcripts under each condition of the DH and the RH treatment (1.2–2.16 fold more), whereas the opposite was true for the desiccation sensitive *S. denticulata* with more repressed than induced transcripts during DH (1.12–1.35 fold more). The other tolerant species, *S. sellowii*, presented a similar pattern to *S. lepidophylla* but only during the initial stages of DH (70 and 50%, with 1.13 and 2.05 fold more induced than repressed), but at extreme DH (10%) and RH times the majority of transcripts were repressed (1.04 to 2.05 fold more repressed than induced). *S. lepidophylla* and *S. sellowii* evolved from a common ancestor adapted to arid environments [1], it could be expected that they share some common DT mechanisms if the ancestor had the ability to survive desiccation. However, transcriptome analysis suggested that these two *Selaginella* species evolved distinct strategies to acquire DT. Comparative analysis of responsive orthogroups in each species showed only 72 protein families induced in both tolerant and sensitive species during DH, corresponding to less than 8% of the total response in each species. Tolerant species shared a higher number of induced orthogroups with around 25 and 10% of the total DH and RH responsive groups, respectively. These findings do not support the notion that the majority of DT responses are common or conserved among these tolerant species, but rather that activation or repression of different sets of genes occurs during the desiccation process in tolerant species. For example, LEA proteins accumulate to high levels during water deficit and have an important protective role in seed and vegetative DT [17, 46], but the same LEA genes are not similarly expressed in both tolerant *Selaginella* species. According to homology analysis, orthogroup OG2082 contains a single copy LEA protein gene per species, but this gene was induced by DH and RH in *S. lepidophylla* but not in *S. sellowii* or *S. denticulata*. This observation suggests that genes present in all three *Selaginella* species could have evolved expression responses to desiccation that are specific for each species. These findings suggest a possible event of convergent evolution in the acquisition of DT in closely related *Selaginella* species.

Homology analysis showed that most of the induced orthogroups in tolerant species were also present in the sensitive species. Previous hypotheses suggested that DT evolved from genetic components also present in sensitive plant species [9, 47–49], indicating that DT arose by rewiring of regulatory networks rather than the acquisition of novel genes. Evidence of differential regulation between tolerant and sensitive *Selaginella* species is shown by the expression patterns of PLATZ1, a transcription factor identified as one of the major regulators of seed DT, whose constitutive expression has been demonstrated to increase drought tolerance in vegetative tissues of *Arabidopsis* [40]. Although PLATZ1 in conjunction with other transcription factors is known to regulate the acquisition of DT in seeds [40], this is the firsts report of upregulation of PLATZ1 during DT in vegetative tissues. The induction and regulatory role of PLATZ1 needs to be confirmed in other desiccation tolerant species and in reproductive structures such as spores in order to shed light on the evolution of DT in relation to different plant tissues.

The comparative analysis of this study focused on the DH and RH response separately to obtain a global overview of the desiccation process in these species. Regardless of the small proportion of shared responses to desiccation, functional analysis during DH and RH indicated that *S. sellowii* and *S. lepidophylla* employ similar mechanisms to achieve DT. Enriched categories in tolerant species in comparison to the sensitive species include amino acid and secondary metabolism, redox and transport. Amino acid metabolism was activated as a response to desiccation, where both *S. sellowii* and *S. lepidophylla* induced more genes related to synthesis than degradation, whereas the sensitive species displayed the opposite pattern. Metabolic studies in *S. lepidophylla* detected accumulation of nitrogen-rich amino acids in the dry state, probably as a nitrogen reservoir for the rehydration stage or as precursors to produce protective compounds such as glutathione [50]. Indeed, nitrogen metabolism resulted highly and significantly enriched only during RH of *S. lepidophylla*. Glutathione in addition to ascorbate, peroxiredoxin and thioredoxin (compounds classified as important components of antioxidant defense systems) were also induced in tolerant species during DH. Enzymatic components of the antioxidant defense, specifically dismutases and catalases were highly enriched in *S. lepidophylla*. An increase in these enzymatic activities as tissues lose water has been reported in other *Selaginella* species such as *S. tamariscina* [51]. Notably, redox metabolism remained very active during RH stage in *S. lepidophylla*. Subcategories of secondary metabolism were mostly enriched in flavonoid and phenylpropanoid pathways, also considered to be key components of the antioxidant system. Several types of flavonoids and phenylpropanoids have been reported in *Selaginella* [7] and shown to be more abundant in the dry state of *S. lepidophylla* in comparison to *S. moellendorffii* [13]. Specifically, the phenylpropanoid category included a significant enrichment of genes related to the synthesis of lignin, a polymer with a putative role in the curling and mechanical response of *S. lepidophylla* stems [16, 43].

The transport subcategory “MIPs” was highly enriched in the tolerant species in comparison to *S. denticulata*. These proteins form membrane channels to facilitate transport of water and small solutes across membranes and have a fundamental role in water relations. An EST analysis found a TIP gene as the seventh most abundant transcript during dehydration of *S. lepidophylla* [52]⁠, highlighting the importance of MIPs in DT. Our transcriptome data show that tolerant *Selaginella* species induced mainly PIP and TIP subfamilies and specifically in *S. lepidophylla* MIPs remained active during the RH stage. The genome of the sensitive species *S. moellendorffii* encodes 19 MIPs, including NIP, TIP, PIP, SIP, XIP and HIP subfamilies [53]⁠. Transcriptome assemblies were only classified for PIP, TIP, NIP and SIP subfamilies, therefore a more detailed analysis is needed to define the type and number of MIPs in the species analyzed in the present study.

Some functions were significantly enriched in a single species. For example, ultrastructural studies in *S. lepidophylla* have demonstrated the cell wall is highly folded in the dry state [54], an essential requirement to maintain structural integrity during desiccation. Recently, structural characterization of another tolerant species (*S. involvens*) determined that desiccation also induces substantial modifications in the composition of cell wall [12]⁠. Our transcriptome data corroborate a reorganization of the *S. lepidophylla* cell wall in response to desiccation. Subcategories during DH were mainly represented by cell wall modification and precursor synthesis, the former included xyloglucan endotransglucosylases/hydrolases (XTH) and expansins associated with cell wall loosening and re-assembling [41]. Additionally, the expression of arabinogalactan proteins (AGPs) during water loss could contribute to increase the flexibility of cell walls [55]. A significant number of genes directly involved in cell wall composition were induced during DH including cellulose and hemicellulose synthesis, and pectin esterases. The most enriched processes during RH were: precursor synthesis, modification and cell wall proteins. In particular, AGPs remained induced during RH. An important difference between DH and RH stages in *S. lepidophylla* was an increase in the number of genes in the cell wall degradation subcategory during RH. Although cell wall modification is essential to survive desiccation, this category was not significantly enriched in *S. sellowii*. However, tolerant species induced a higher proportion of pectin esterases during DH in comparison to the sensitive one. The activity of pectin esterases could modify cell wall structure [41] and qRT-PCR analysis corroborated the upregulation of a pectin methylesterase inhibitor in both tolerant species indicating that this mechanism is also present in *S. sellowii*. Although a lower number of enriched genes was observed in the cell wall modification category in *S. sellowii*, similarities with *S. lepidophylla* such as induction of precursors and hemicellulose synthesis were observed. However, *S. sellowii* showed a contrasting pattern to *S. lepidophylla* in the degradation subcategory with more genes differentially expressed during DH than in RH. Taken together, these results strongly suggest that cell wall reorganization plays an important role during the desiccation process in tolerant species.

In response to dehydration all desiccation tolerant plants repress photosynthetic activity [56] and down regulation of photosynthesis-related genes has been reported in several homoiochlorophyllous tolerant species [57–59]. Functional analysis showed an unexpected finding in *S. sellowii*, since during DH a significant number of genes related to Calvin cycle and light-reactions are induced, whereas *S. lepidophylla* and *S. denticulata* displayed a similar number of induced and repressed genes involved in these processes. Net photosynthesis measurements and maximum quantum efficiency of PSII showed a faster recovery of activity of *S. sellowii* explants during RH that could be associated with the induction of photosynthesis-related genes during water loss. Accumulation of photosynthesis related transcripts and/or proteins would provide an ecological advantage for the short time periods during which *S. sellowii* is metabolically active allowing a rapid recovery during rehydration. Furthermore, the rapid activation of photosynthesis shown by *Selaginella* species during RH indicates an effective protection of photosynthetic machinery during water loss and in the dry state. The combination of morphological changes, increased antioxidant activity and induction of proteins with a protective role, specifically ELIPs, could prevent photo-oxidative damage. Induction of ELIP genes in response to water stress has been observed in desiccation tolerant as well as in sensitive plants [21]. The three *Selaginella* species showed upregulation of the expression of ELIPs during DH. However, the sensitive species *S. denticulata* induced these genes only during the intermediate phase of dehydration (50% water content), whereas tolerant species maintained ELIP expression under extreme DH (10% water content). These results indicate an important difference between desiccation tolerant and sensitive *Selaginella* species where expression of ELIPs at low water content in tolerant species could provide protection against photo-oxidative damage in the dry state that is lacking in sensitive species. Furthermore, ELIP genes continued to be upregulated at early RH (2 h) but were switched off at later RH times. This is consistent with reports for other desiccation tolerant species that show similar patterns of decreased ELIPs expression when plants return to normal water contents during RH [21].

Sugar accumulation during dehydration is one of the principal characteristics of desiccation tolerant plants and carbohydrate metabolism differs widely across different species [60]⁠. High levels of trehalose in hydrated and dehydrated tissues have been reported in several species of the *Selaginella* genus, including sensitive species [13, 61–63]⁠. Previous studies have shown that the first enzyme of trehalose synthesis (TPS) is constitutively expressed in *S. lepidophylla* [64], whereas our transcriptome data indicated that the second enzyme of the synthesis, TPP was induced during dehydration in tolerant species. Although heterologous expression of trehalose genes has improved crop tolerance to abiotic stress [65]⁠, a specific role in DT has not been clearly defined. Raffinose family oligosaccharides (RFOs) are also associated with acquisition of DT in seeds and vegetative tissues in some species, however neither *S. lepidophylla* nor *S. tamariscina* have detectable levels of RFOs (specifically raffinose and stachyose) [50, 62]⁠. In contrast, the *S. sellowii* transcriptome was highly enriched in terms related to raffinose family metabolism specifically during dehydration. These results support the hypothesis that *S. sellowii* employs different strategies for exploiting carbohydrate metabolism in response to desiccation in contrast to other species. Carbohydrate composition analyzed by thin layer chromatography showed a highly variable carbohydrate pattern between *S. sellowii* and *S. lepidophylla* (data not shown), however a more detailed analysis is needed to determine the significance of carbohydrate metabolism in relation to DT in *S. sellowii*.

Tolerant species *S. sellowii* and *S. lepidophylla* are closely related [3–6] and predicted proteomes showed high homology between them. However, transcriptome data showed that although almost all of the desiccation responsive transcripts have orthologues in the other species, they were mainly induced only in one species. In response to DH and RH, both tolerant *Selaginella* species shared the induction of several mechanisms considered as essential to survive desiccation. These results suggest convergent evolution of DT ability in *S. sellowii* and *S. lepidophylla*, probably due to specific rewiring of the regulatory networks orchestrating DT during the adaptation of each of these species to their specific natural habitats. A phylogenetic analysis indicated that a common ancestor of the clades to which these species belong was adapted to arid regions [1], but there is no sufficient evidence to determine if this ancestor was also a desiccation tolerant organism.

**Fig. 7:**
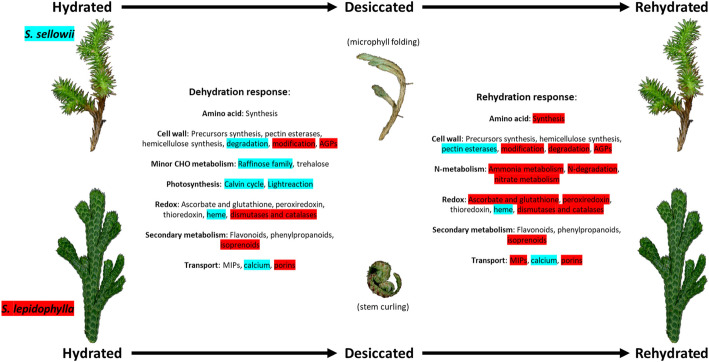
Schematic representation of common and specific responses to desiccation in *S. sellowii* and *S. lepidophylla*. Functional categories significantly enriched in both tolerant *Selaginella* species and species-specific responses (highlighted in blue for *S. sellowii* and in red for *S. lepidophylla*) during dehydration and rehydration. Predominant morphological changes during dehydration are indicated in brackets

Comparative analysis of tolerant *Selaginella* species with very different morphologies and growth forms showed convergence in some of the major responses to water loss such as a significant number of upregulated genes involved in secondary metabolism (flavonoids and phenylpropanois), antioxidant systems (ascorbate, glutathione, peroxiredoxin and thioredoxin), MIPs and amino acid synthesis (Fig. 7). Specific responses also have an important role in the acquisition of DT in each species and could have evolved in response to their particular habitats and adaptations. An example is the induction of photosynthesis related genes during DH in *S. sellowii* leading to faster reactivation of photosynthesis during RH consistent with shortened periods of metabolic activity or the more pronounced cell wall modification response in *S. lepidophylla*. To confirm these hypotheses, more detailed characterization is needed to determine specific patterns and levels of expression of particular gene sets during the desiccation process in tolerant species in comparison to sensitive species.

## Conclusions

The *Selaginella* genus represents a useful model to study the mechanisms involved in the acquisition of vegetative DT in a basal vascular clade. The present study confirmed the DT nature of *S. sellowii* and the facility of analyzing explant samples. Accurate and complete reference transcriptomes adequate for analysis of the molecular aspects of DT and biology of *Selaginella* were developed. Although predicted proteomes share a significant number of protein families, most of the differentially expressed genes under DT between *S. sellowii* and *S. lepidophylla* are distinct despite their phylogenetic relatedness. Tolerant *Selaginella* species with contrasting morphologies showed common as well as specific responses to DH and RH and this is summarized in Fig. 7 and functional analysis suggests convergent evolution in some of the major categories relevant to DT. The comparative analysis with *S. denticulata* allows us to discriminate between DT responses and general drought responses in *Selaginella* species indicating that some of the major differences between tolerant and sensitive species include aspects of amino acid and secondary metabolism during water loss. Both shared and species-specific antioxidant responses were highly represented in tolerant species. Additionally, our results indicate differences between tolerant species in photosynthesis related genes and carbohydrate metabolism during dehydration in *S. sellowii*, and cell wall remodeling and N-metabolism (specifically during RH) in *S.lepidophylla*.

## Methods

### Plant material and drying treatments

*S. sellowii* plants were collected in San Jose del Chilar, Oaxaca, Mexico (17°42′56.2″ N, 96°56′28.4″ W). *S. lepidophylla* were collected in Tlacotepec, Morelos, Mexico (18°49′19.6″ N, 98°45′10.3″ W) (Additional file 12). Both species were maintained in a dried state until analyzed. Specimens above were morphologically identified and deposited at MEXU herbarium (UNAM) by professor Daniel Tejero-Díez from Faculty of Higher Studies (FES) Iztacala, UNAM. *S. denticulata* samples belonging to the Botanical Garden of the Biology Institute, UNAM, were provided by Aída Telléz Velasco. Before desiccation experiments, tolerant species were rehydrated and acclimatized to greenhouse/growth chamber conditions for at least 5 days. Desiccation experiments were carried out in 310 ml pots containing Sunshine®:Tezontle (2:1) under greenhouse conditions. Water was withheld for a month and the pots were weighed every 2 days until reaching a constant weight. The average values of RH (%) and temperature were recorded until a constant weight was reached. To evaluate DT, plants were watered and maintained in a hydrated condition for at least 1 week. Drying rates were established using explants from well-watered individuals, removing nonviable tissue and washing twice with distilled water; *S. lepidophylla* explants were collected from the 3rd and 4th rows of its circular arrangement. Explants were kept floating on water for 30 min in a growth chamber at 24 °C, excess water was then removed by blotting and explants were placed in Petri dishes for weighing (W_i_). Samples were then immediately incubated in a growth chamber (24 °C) inside closed plastic boxes (40 × 21.5 × 13 cm) containing a saturated salt solution (MgCl_2_) to lower the humidity of the system. Samples were removed for weighing at regular intervals (W_x_) over a 24 h period. Following the desiccation period, samples for rehydration were submerged in deionized water for specific times. The water content (WC) was calculated using the following formula: WC = (W_x_ – W_d_)/(W_i_ – W_d_) × 100, where W_d_ is the dry weight obtained after incubation at 80 °C for 15 h.

### RNA extraction, library construction and sequencing

Explants were obtained from fully hydrated tissue, at specific time points during dehydration (70, 50 and 10% of water content determined using the drying curves), and time points during rehydration (tolerant species). RNA was extracted using PureLink™ Plant RNA Reagent (Invitrogen), and treated with DNase I (Roche), then further purified using TRIzol™ Reagent (Invitrogen). Integrity of RNA samples was assessed using the Agilent 2100 Bioanalyzer (Agilent Technologies). RNA from two independent replicates was pooled for library preparation using TruSeq RNA Library Prep Kit v2 (Illumina). cDNA libraries were sequenced using the 2 × 75 paired-end mode on the NextSeq platform (Illumina). Libraries and sequencing were performed at the Laboratory of Genomic Services (UGA-LANGEBIO, Cinvestav, Mexico).

### De novo transcriptome assembly and annotation

Raw data was preprocessed with Trimmomatic (v0.35) [66]⁠ to remove adapter sequences and filter low quality reads. De novo transcriptome assemblies were obtained using Trinity (v2.1.1) [35]⁠ and SOAPdenovo-Trans (v1.04) [36]⁠ with default parameters. Quality assessment of the resulting assemblies included: rnaQUAST (v1.4.0) [67]⁠, BUSCO (v2.0); eukaryotic subset) [37]⁠ and DETONATE (v1.11) [45]. Selected transcriptomes were functionally annotated using BLASTX (bitscore ≥90) against plant RefSeq (NCBI) and UniProt/SwissProt databases, and protein models of the following species: *A. thaliana*, *S. moellendorffii*, *Physcomitrella patens*, and *Amborella trichopoda* (Ensembl Plants). All transcriptome versions (different software and reference set) and annotation are available on request from the authors.

### Identification of responsive transcripts and functional enrichment analysis

Reference transcriptomes were used to quantify the expression in the different hydration states. Alignment of the trimmed reads was performed using Bowtie2 [68]⁠ and RSEM [69]⁠ for transcript abundance estimation. Expression levels were quantified with edgeR [70]. To calculate dispersion parameter for each species, a set of transcripts with low specificity across libraries was classified as housekeeping genes according to an algorithm developed by Martinez and Reyes-Valdés [71]. Transcripts with a logFC ≥1 or ≤ − 1 and FDR < 0.01 were considered as significant. Functional categories analysis was carried out using MapMan terms in the SuperViewer online server (http://bar.utoronto.ca/) by submitting the transcripts with their *A. thaliana* annotation [72]. MapMan classification were obtained for reference assemblies and for differentially expressed transcripts in response to dehydration or rehydration. Categories in response to dehydration or rehydration with a *p*-value < 0.05 (SuperViewer analysis) were classified as significantly enriched categories. Each category was expressed as the ratio of the induced category (absolute values) over the total number of the same categories represented in the reference transcriptome assembly

### Proteome prediction and clustering

Protein sequences were predicted using TransDecoder (https://transdecoder.github.io/, v2.0.1) by filtering with a minimum length of 100 amino acids. Orthogroups of predicted *Selaginella* proteins were clustered according to the OrthoFinder algorithm (v2.1.2) [39]⁠ using default settings. The Venn diagram tool jvenn [73]⁠ was used to compare orthogroups between species.

### Quantitative real-time PCR analysis

Single copy orthogroups that resulted differentially induced in tolerant species were selected for validation. The qRT-PCR reactions were performed for hydrated explants (control condition), extreme dehydration (10% water content) and early rehydration (2 h). The qRT-PCR was carried out in a Magnetic Induction Cycler (Biomolecular Systems) using species-specific or universal PCR primers (information is provided in Additional file 5) and reagent SensiFast TM SYBR No-ROX kit (Bioline). The PCR cycling conditions were: 95 °C for 2 min, followed by 40 cycles of 95 °C for 5 s, 65 °C for 10 s and 72 °C for 20 s. Expression levels were calculated relative to the reference gene (ubiquitin protein ligase) using the formula 2^(−ΔCT)^. Fold change values in comparison to the control condition per species.

### Photosynthetic parameters

Gas exchange measurements were carried out with a CIRAS-3 portable photosynthesis system (PP Systems, USA). Net photosynthetic rate in explants was determined with a cuvette flow of 250 cc min^− 1^, CO_2_ concentration at 390 μmol mol^− 1^, 70% of relative humidity, and light intensity at 1000 μmol m^− 2^ s^− 1^. Data were dry mass normalized to compare between species. Fv/Fm was determined in 15 min dark adapted explants using a Pocket PEA chlorophyll fluorimeter (Hansatech Instruments, UK).

## Supplementary information

**Additional file 1: Figure S1.** Desiccation tolerance (DT) capacity of explants and drying rates of *S. lepidophylla* and *S. denticulata*.

**Additional file 2: Figure S2.** Morphology and RNA integrity of *S. lepidophylla* and *S. denticulata* during the desiccation process.

**Additional file 3: Figure S3.** Size distribution of desiccation responsive transcripts and their annotation.

**Additional file 4: Figure S4.** Comparison of protein sequence identity between *Selaginella* species.

**Additional file 5: Figure S5.** Quantitative real-time PCR validation.

**Additional file 6: Figure S6.** Subcategories of induced genes during the dehydration process.

**Additional file 7: Figure S7.** Functional analysis and subcategories of rehydration induced genes.

**Additional file 8: Figure S8.** Metabolism of photosynthesis during rehydration in tolerant species.

**Additional file 9: Table S1.** List of subcategories of the MapMan analysis.

**Additional file 10: Table S2.** Subfamilies of major intrinsic proteins (MIPs) responsive to desiccation in *Selaginella*.

**Additional file 11: Table S3.** Cell wall subcategories induced during DH and RH.

**Additional file 12.** Morphological identification, habitat description and coordinates.

## Data Availability

Raw data generated in this study have been deposited in the NCBI Sequence Read Archive (SRA) repository with accessions SRR11554869 to SRR11554884. Trinity de novo transcriptome assemblies have been deposited at DDBJ/EMBL/GenBank Transcriptome Shotgun Assembly (TSA) under the accessions GIMF00000000, GIMG00000000 and GIMH00000000.

## Abbreviations

AGPs: Arabinogalactan proteins
BUSCO: Benchmarking Universal Single-Copy Orthologs
DH: Dehydration
DT: Desiccation Tolerance
ELIPs: Early Light-Induced Proteins
LEA: Late Embryogenesis Abundant
MIPs: Major Intrinsic Proteins
RFOs: Raffinose Family Oligosaccharides
RH: Rehydration
ROS: Reactive oxygen species
TPP: Trehalose-6-phosphate phosphatase
TPS: Trehalose phosphate synthase
XTH: Xyloglucan endotransglucosylases/hydrolases
