# Supplementary material for: Comparative transcriptome analysis suggests convergent evolution of desiccation tolerance in Selaginella species
Source: BMC Plant Biol. 2020 Oct 12;20:468. doi: 10.1186/s12870-020-02638-3 (PMC7549206; doi:10.1186/s12870-020-02638-3)
Supplement: Supplementary file 1 — Additional file 1: Figure S1. Desiccation tolerance (DT) capacity of explants and drying rates of S. lepidophylla and S. denticulata. [file 12870_2020_2638_MOESM1_ESM.pdf]

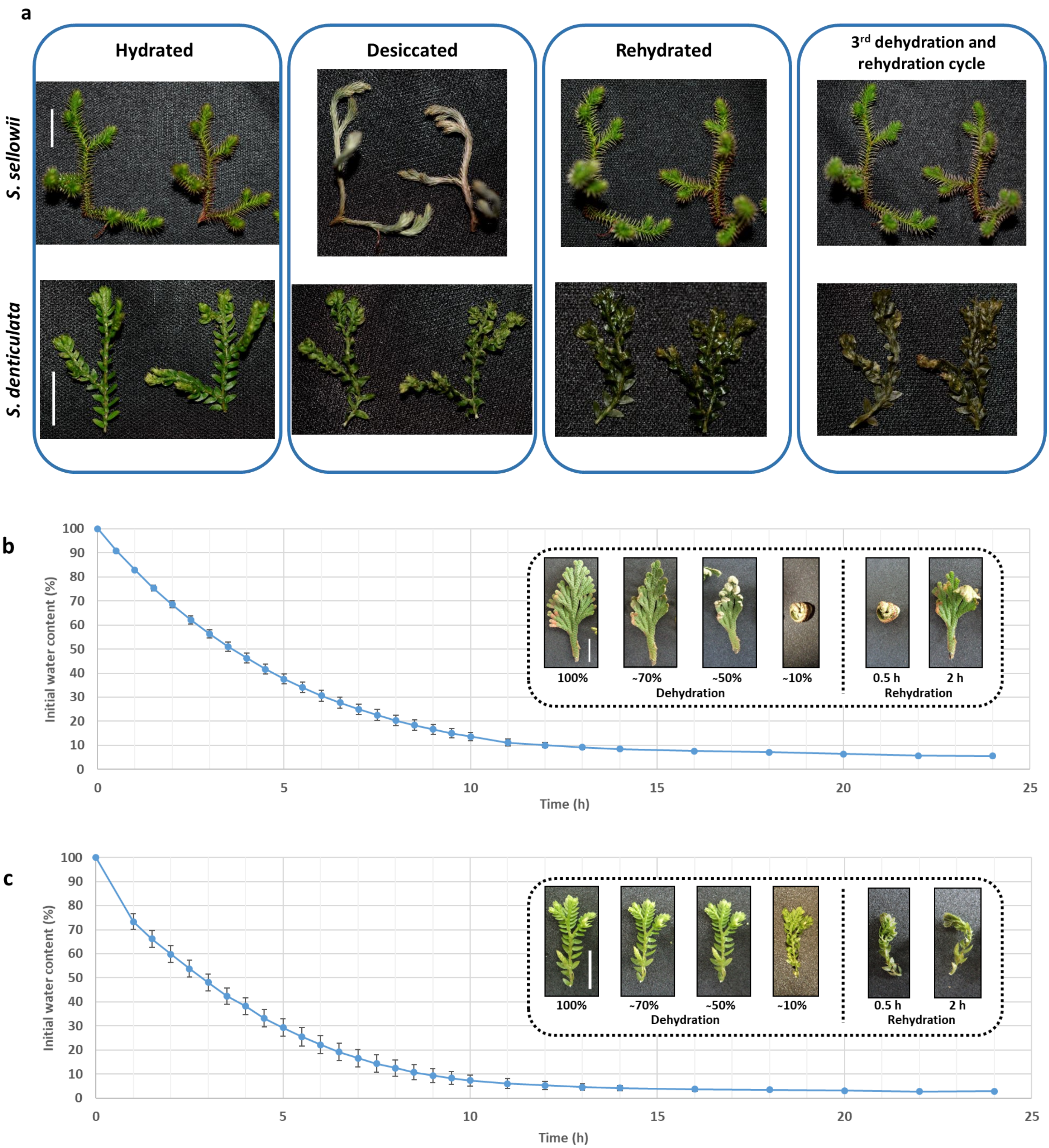

Figure S1. Desiccation tolerance (DT) capacity of explants and drying rates of *S. lepidophylla* and *S. denticulata*. (**a**) Morphologies of explants exposed to repeated dehydration and rehydration cycles. (**b**) Examples of the changes in morphology of *S. denticulata* and (**c**) *S. lepidophylla* explants during dehydration (loss of initial water content) and rehydration. Points represent mean values of 4 replicates ± SD. Scale bar 1 cm.
