# Supplementary material for: Comparative transcriptome analysis suggests convergent evolution of desiccation tolerance in Selaginella species
Source: BMC Plant Biol. 2020 Oct 12;20:468. doi: 10.1186/s12870-020-02638-3 (PMC7549206; doi:10.1186/s12870-020-02638-3)
Supplement: Supplementary file 2 — Additional file 2: Figure S2. Morphology and RNA integrity of S. lepidophylla and S. denticulata during the desiccation process. [file 12870_2020_2638_MOESM2_ESM.pdf]

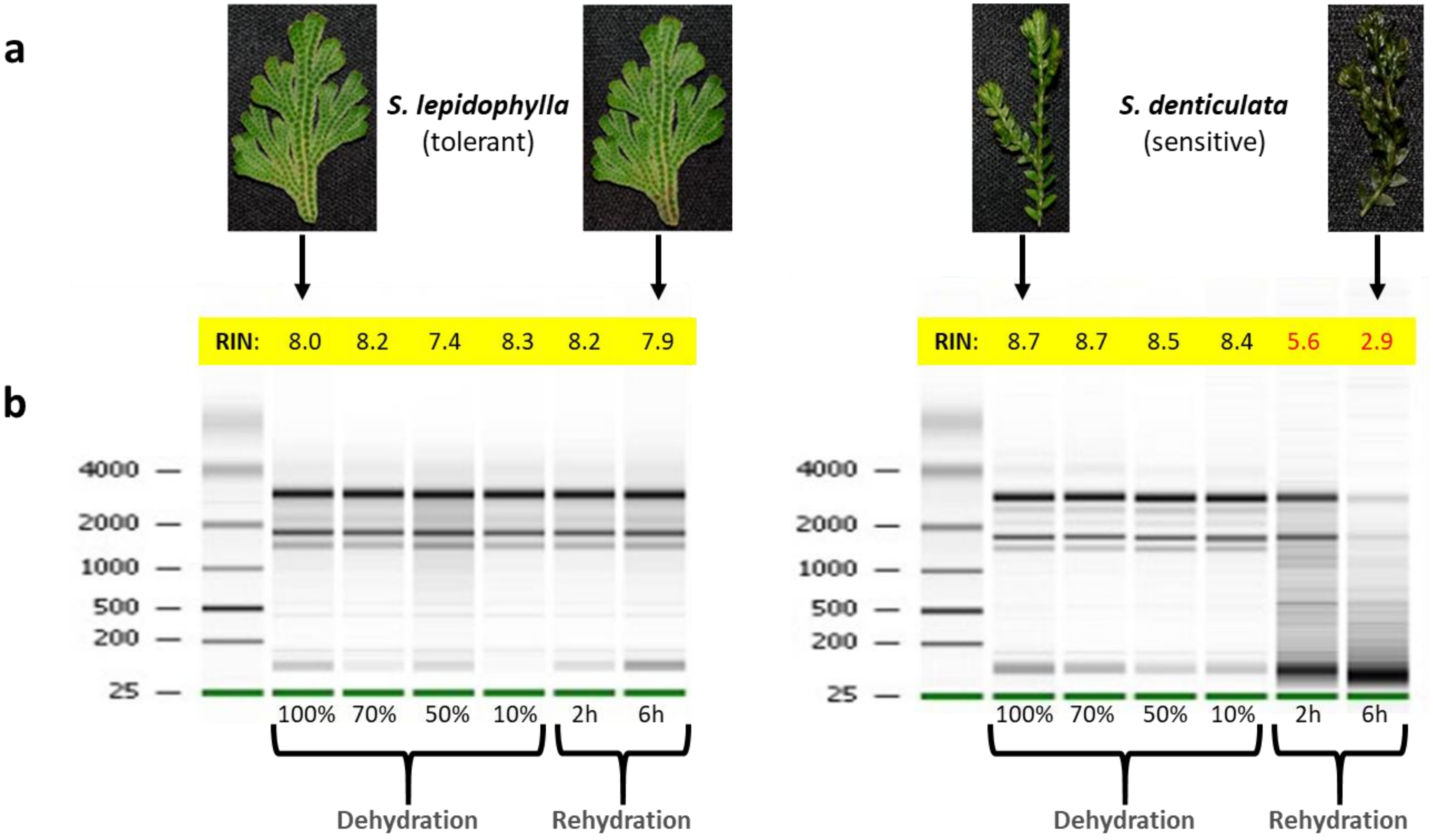

Figure S2. Morphology and RNA integrity of *S. lepidophylla* and *S. denticulata* during the desiccation process.
(**a**) Representative images of explants in hydrated and rehydrated conditions. (**b**) RNA profiles obtained with the Agilent Bioanalyzer System, intense bands correspond to 18S and 25S rRNA. RNA integrity expressed as RIN values. Numbers in red indicate significant RNA degradation. Water contents (%) or rehydration times are shown below each image.
