# Supplementary material for: Comparative transcriptome analysis suggests convergent evolution of desiccation tolerance in Selaginella species
Source: BMC Plant Biol. 2020 Oct 12;20:468. doi: 10.1186/s12870-020-02638-3 (PMC7549206; doi:10.1186/s12870-020-02638-3)
Supplement: Supplementary file 3 — Additional file 3: Figure S3. Size distribution of desiccation responsive transcripts and their annotation. [file 12870_2020_2638_MOESM3_ESM.pdf]

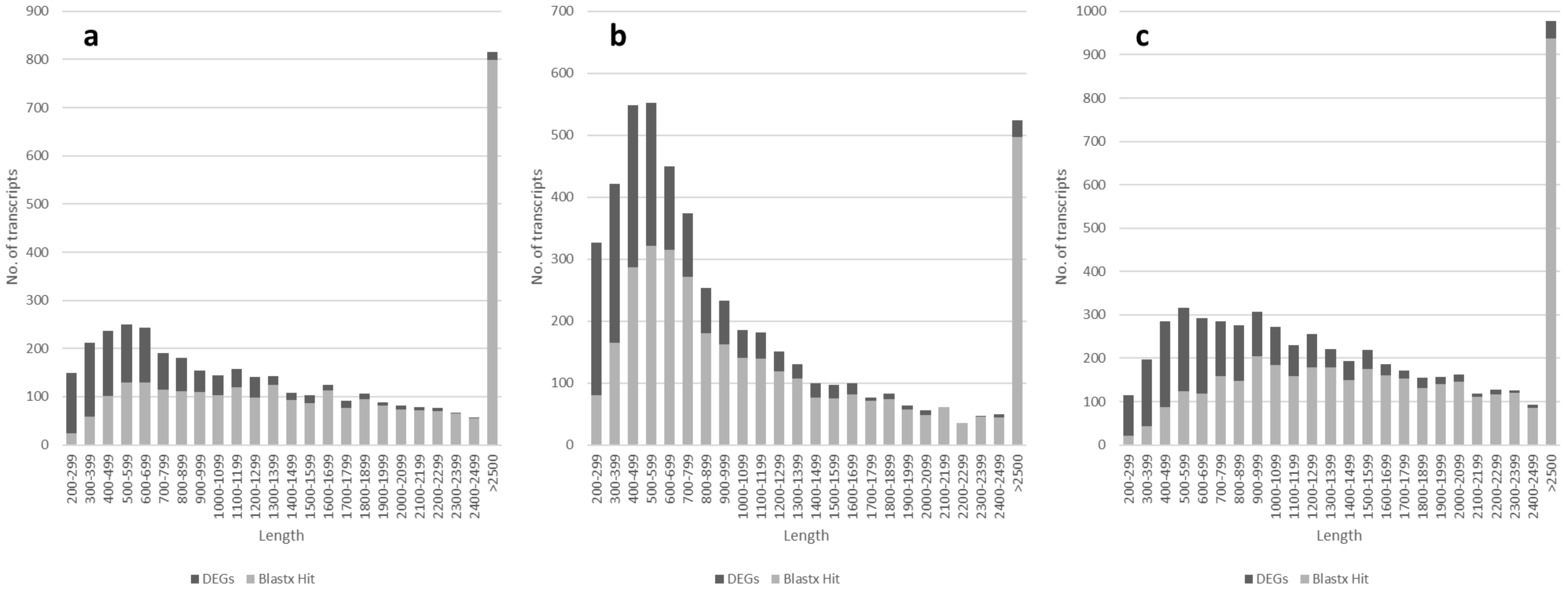

Figure S3. Size distribution of desiccation responsive transcripts and their annotation.
Proportion of desiccation responsive transcripts with annotation (Blastx) in different databases and gene models for (**a**) *S. sellowii*, (**b**) *S. lepidophylla* and (**c**) *S. denticulata*.
