# Supplementary material for: Comparative transcriptome analysis suggests convergent evolution of desiccation tolerance in Selaginella species
Source: BMC Plant Biol. 2020 Oct 12;20:468. doi: 10.1186/s12870-020-02638-3 (PMC7549206; doi:10.1186/s12870-020-02638-3)
Supplement: Supplementary file 4 — Additional file 4: Figure S4. Comparison of protein sequence identity between Selaginella species. [file 12870_2020_2638_MOESM4_ESM.pdf]

a

RBH (mean identity):

Subject / Query

	S. sellowii	*S. lepidophylla*	*S. denticulata*
S. sellowii	---	71.12 %	66.52 %
S. lepidophylla	71.15 %	---	65.31 %
S. denticulata	66.52 %	65.30 %	---

b

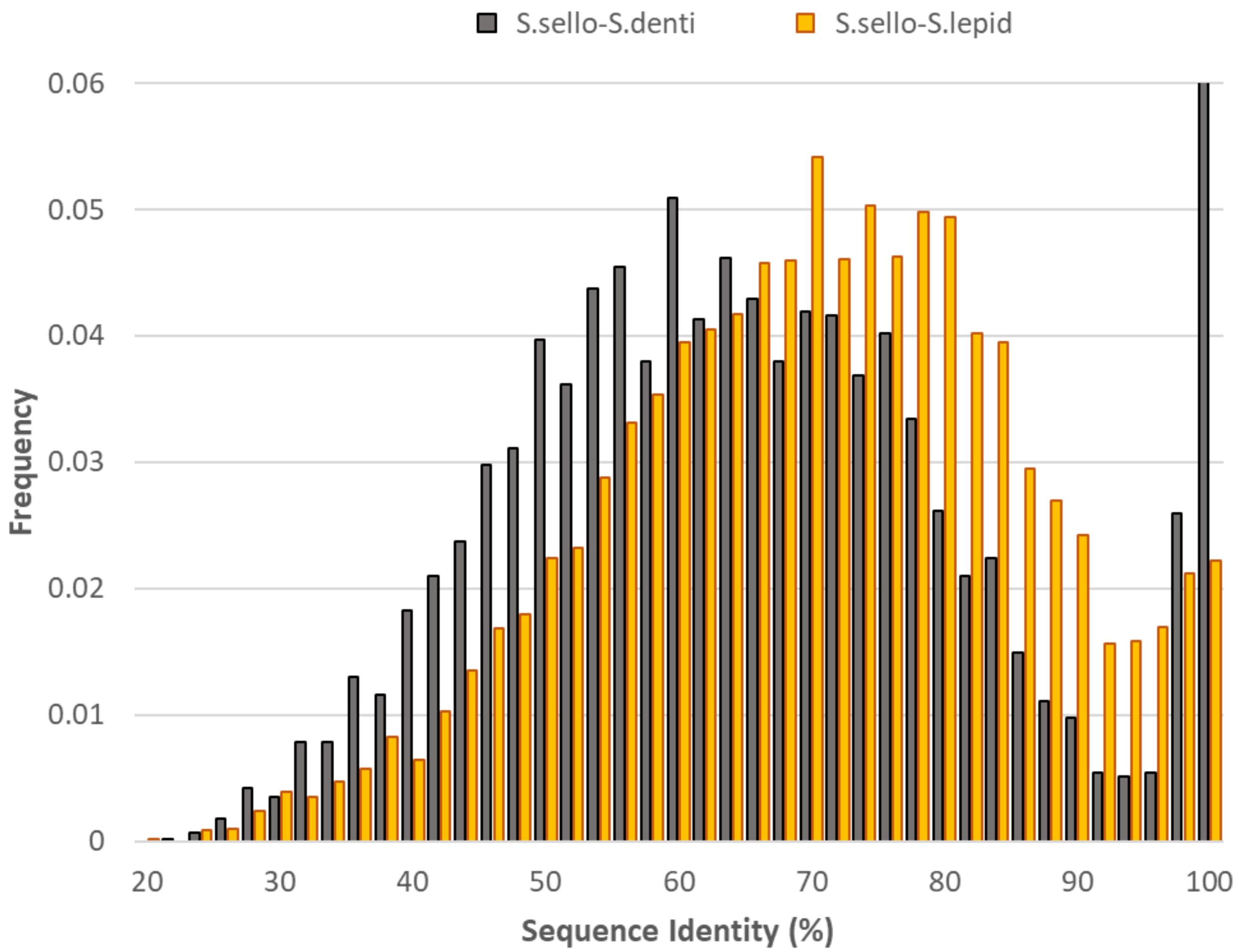

Figure S4. Comparison of protein sequence identity between *Selaginella* species.
(**a**) Reciprocal best hits (RBH) expressed as an average percentage identity. (**b**) Pairwise comparisons of frequencies of shared protein identities between *S. sellowii* (S. sello) and *S. lepidophylla* (S. lepid) and *S. sellowii* (S. sello) and *S. denticulata* (S. denti) species.
