# Supplementary material for: Comparative transcriptome analysis suggests convergent evolution of desiccation tolerance in Selaginella species
Source: BMC Plant Biol. 2020 Oct 12;20:468. doi: 10.1186/s12870-020-02638-3 (PMC7549206; doi:10.1186/s12870-020-02638-3)
Supplement: Supplementary file 5 — Additional file 5: Figure S5. Quantitative real-time PCR validation. [file 12870_2020_2638_MOESM5_ESM.pdf]

a

Orthogroup	Species	Forward primer	Reverse primer
OG5138	*S. sellowii, S. lepidophylla*	GTCTTCCTACCACGACGTAGT	CGGTCGCATATCTCGCAAGTCT
	S. denticulata	CGCAGCACCGTGATCACCACGT	
OG4174	*S. sellowii*	CTGTGGACTTCATCTTCGGCA	TGAAGACGGGCAGGAAGTC
	S. lepidophylla	CCGTGGACTTCATCTTTGGGA	GAAGATCGGAAGGTAGTCCCG
	S. denticulata	TTGGCAATGCAGCTTCGGTA	GACGATGGGAAGGAAGTCCG
OG2082	*S. sellowii*	CTACAAGGACATCGAGGAGACG	GAGAGATCGCAGGACAAGTGCT
	S. lepidophylla	CAAGACATCGAGGACACATACG	AGATCGCAGCTAAGATGCTTCA
	S. denticulata	CTACGACGACATCGAGAATACG	GCTCAAGATCATACTGGAGCTC
OG3782	*S. sellowii, S. lepidophylla*	TGGCTCGTCAAATGCAAGATG	TACTGCCTTCTCGTCTCGAA
	S. denticulata		TACTGCCGTCTCGTCTCGAA

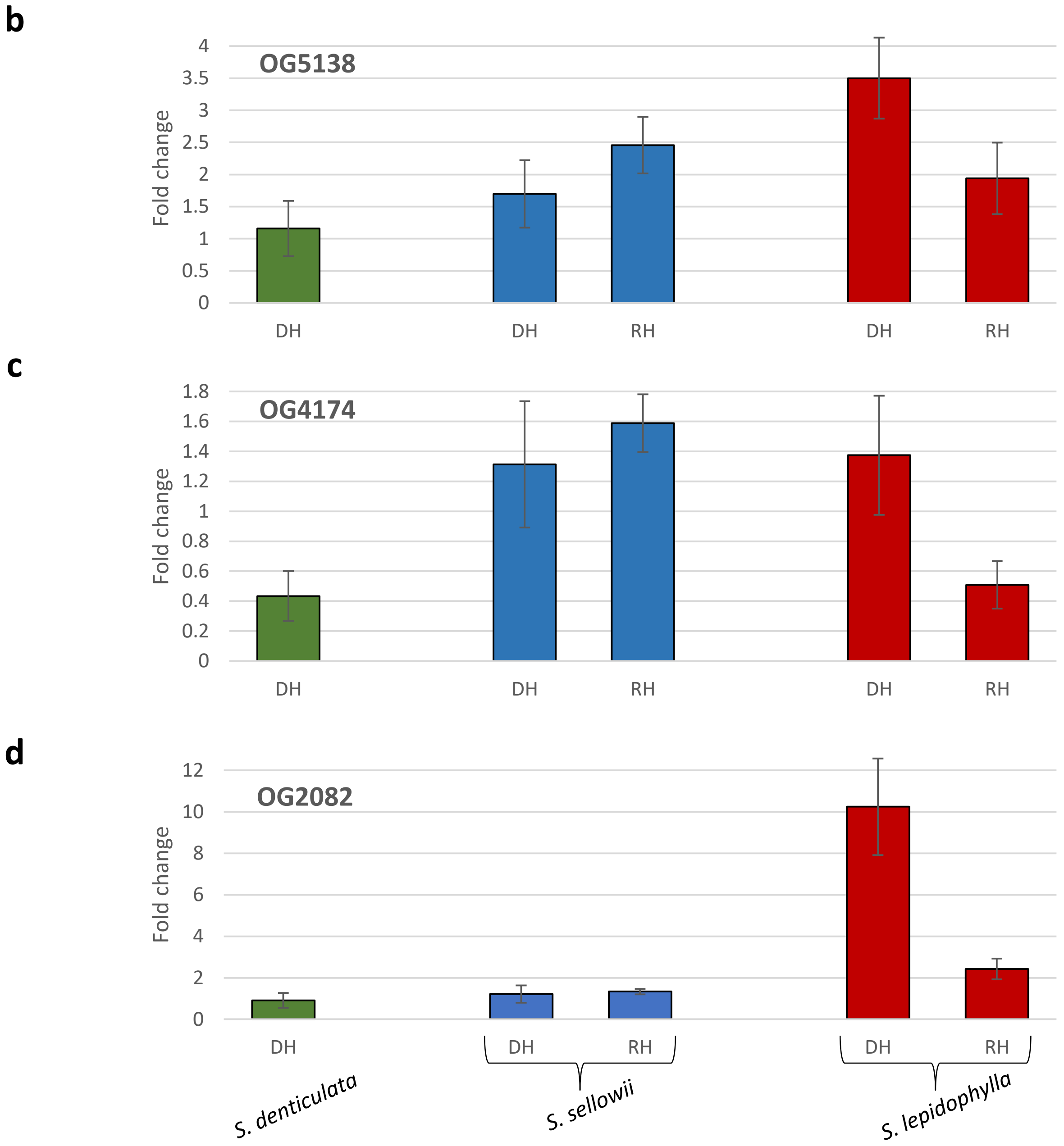

Figure S5. **Quantitative real-time PCR validation.** (**a**) Selected orthogroups and primers used for qRT-PCR analysis. Expression levels in *S. denticulata* (green), *S. sellowii* (blue) and *S. lepidophylla* (red) of the orthogroups (**b**) OG5138, (**c**) OG4174 and (**d**) OG2082. An ubiquitin protein ligase gene (OG3782) was used as reference gene. Fold change with respect to hydrated conditions in response to DH (extreme dehydration; 10% water content) or RH (early rehydration; 2 h). Data are shown as the mean and standard error of two biological replicates and three technical replicates for each sample.
