# Supplementary material for: Comparative transcriptome analysis suggests convergent evolution of desiccation tolerance in Selaginella species
Source: BMC Plant Biol. 2020 Oct 12;20:468. doi: 10.1186/s12870-020-02638-3 (PMC7549206; doi:10.1186/s12870-020-02638-3)
Supplement: Supplementary file 6 — Additional file 6: Figure S6. Subcategories of induced genes during the dehydration process. [file 12870_2020_2638_MOESM6_ESM.pdf]

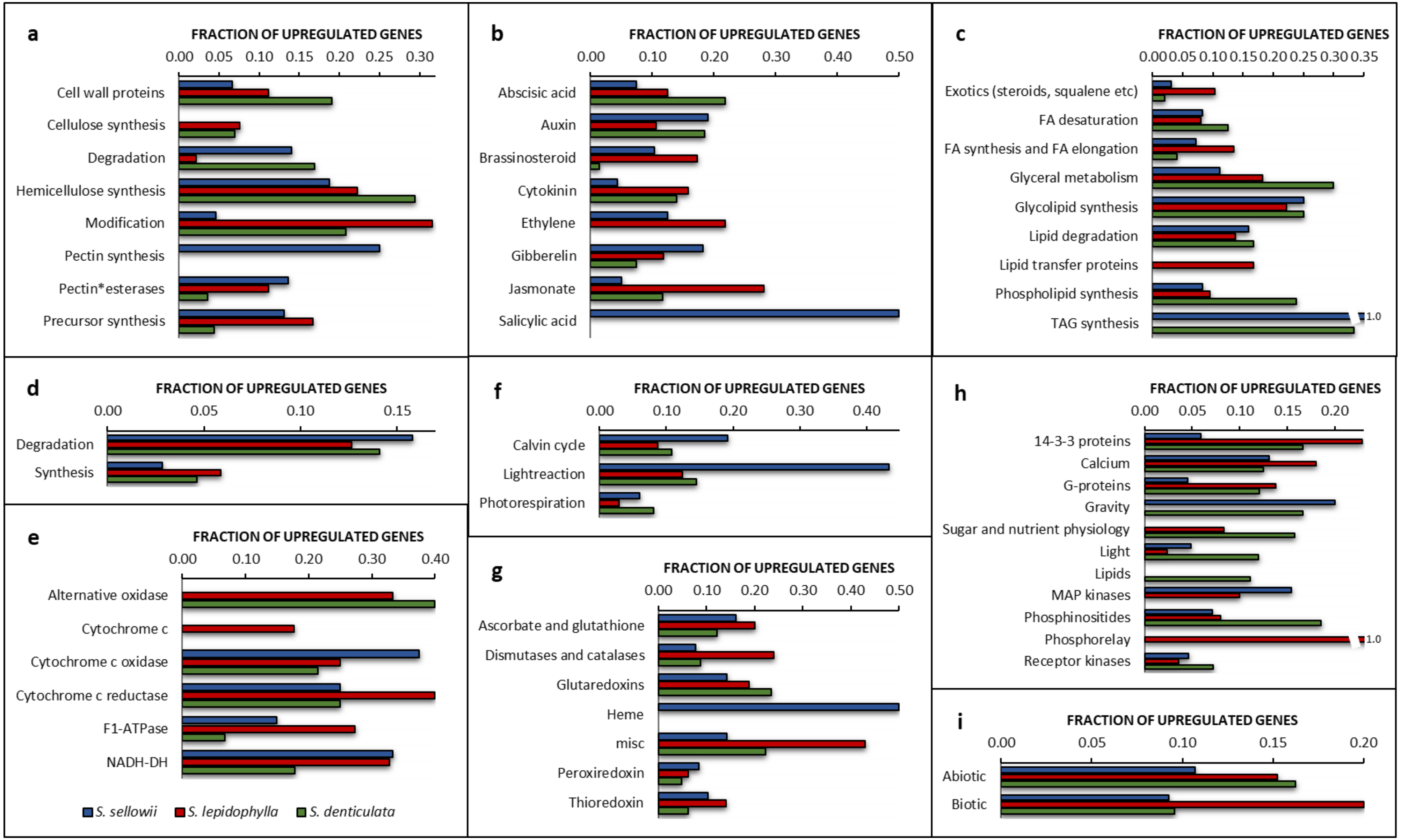

Figure S6. Subcategories of induced genes during the dehydration process.
Subcategories: (**a**) cell wall, (**b**) hormone, (**c**) lipid, (**d**) major CHO metabolism, (**e**) mitochondrial e- transport/ATP, (**f**) photosynthesis, (**g**) redox, (**h**) signaling, and (**i**) stress.
