# Supplementary material for: Comparative transcriptome analysis suggests convergent evolution of desiccation tolerance in Selaginella species
Source: BMC Plant Biol. 2020 Oct 12;20:468. doi: 10.1186/s12870-020-02638-3 (PMC7549206; doi:10.1186/s12870-020-02638-3)
Supplement: Supplementary file 7 — Additional file 7: Figure S7. Functional analysis and subcategories of rehydration induced genes. [file 12870_2020_2638_MOESM7_ESM.pdf]

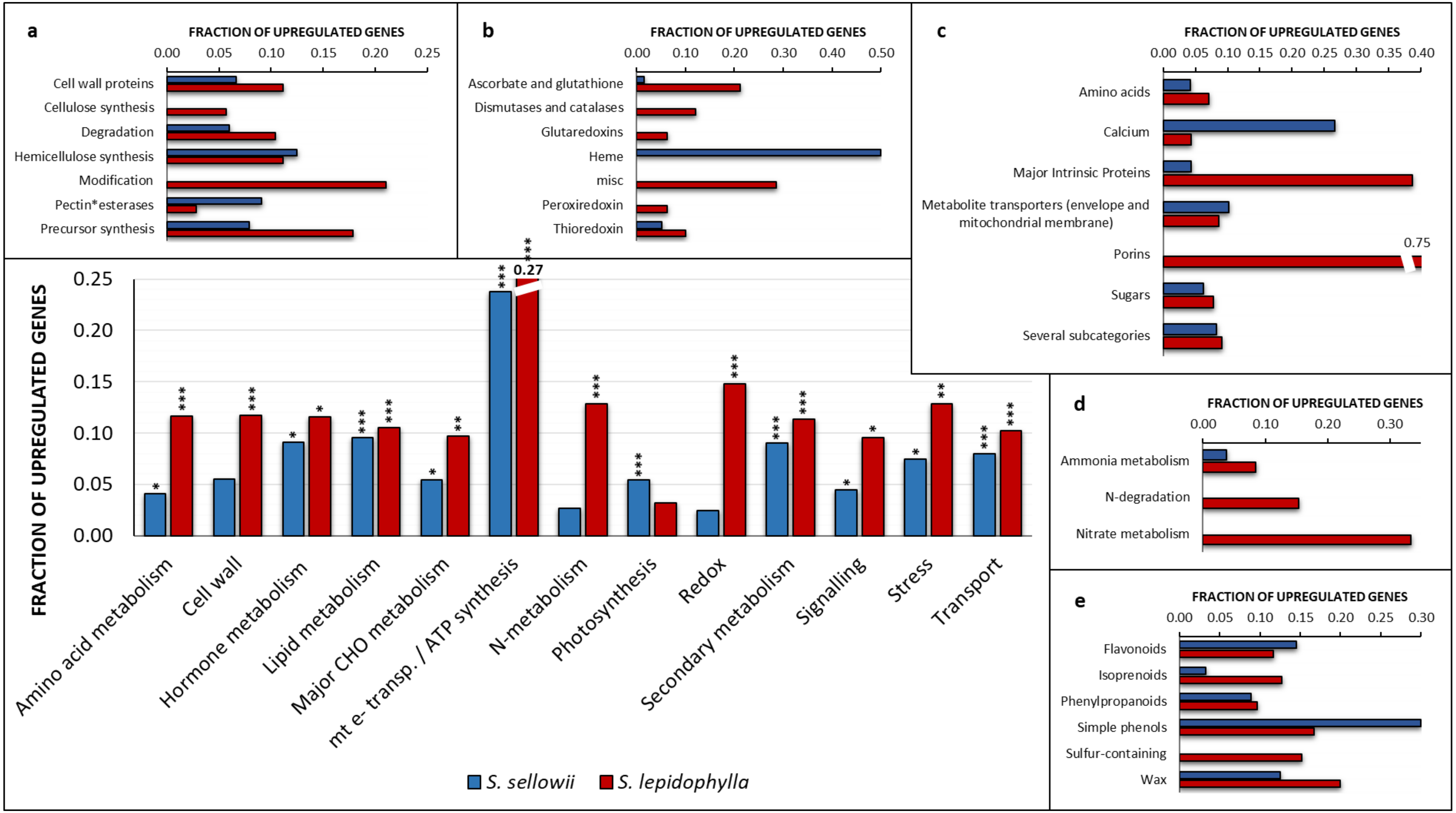

Figure S7. Functional analysis and subcategories of rehydration induced genes.
Subcategories: (**a**) cell wall, (**b**) redox, (**c**) transport, (**d**) N-metabolism and (**e**) secondary metabolism.
