# Supplementary material for: Comparative transcriptome analysis suggests convergent evolution of desiccation tolerance in Selaginella species
Source: BMC Plant Biol. 2020 Oct 12;20:468. doi: 10.1186/s12870-020-02638-3 (PMC7549206; doi:10.1186/s12870-020-02638-3)
Supplement: Supplementary file 8 — Additional file 8: Figure S8. Metabolism of photosynthesis during rehydration in tolerant species. [file 12870_2020_2638_MOESM8_ESM.pdf]

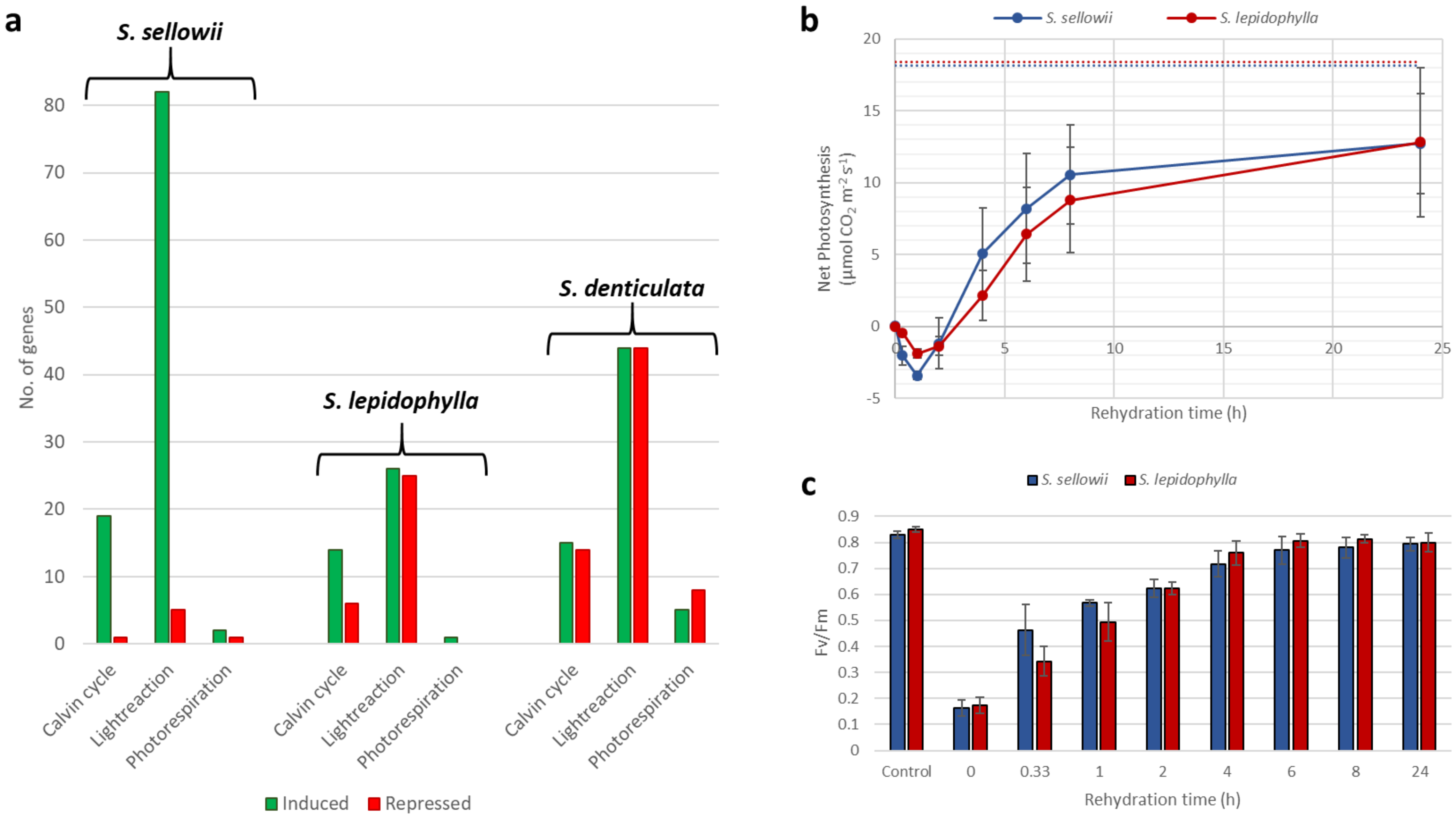

Figure S8. Metabolism of photosynthesis during rehydration in tolerant species.
(**a**) Numbers of photosynthesis associated genes induced or repressed during DH in the 3 *Selaginella* species analyzed. (**b**) Time course of the recovery of photosynthetic activity during RH of *S. sellowii* and *S. lepidophylla* explants. Dotted lines indicate photosynthesis levels of healthy unstressed tissue. (**c**) Maximum quantum efficiency of PSII (Fv/Fm). Points represent mean values of 3 replicates ± SD.
