# Supplementary material for: Comparative transcriptome analysis suggests convergent evolution of desiccation tolerance in Selaginella species
Source: BMC Plant Biol. 2020 Oct 12;20:468. doi: 10.1186/s12870-020-02638-3 (PMC7549206; doi:10.1186/s12870-020-02638-3)
Supplement: Supplementary file 10 — Additional file 10: Table S2. Subfamilies of major intrinsic proteins (MIPs) responsive to desiccation in Selaginella. [file 12870_2020_2638_MOESM10_ESM.pdf]

Table S2. Subfamilies of major intrinsic proteins (MIPs) responsive to desiccation in *Selaginella*.

Species	HIP	XIP	SIP	PIP	TIP	NIP	Total
S. moellendorffii	**2**	**3**	**1**	**3**	**2**	**8**	19 genes*
S. sellowii	-	-	-	**9** (3/0)	**7** (2/0)	**7** (1/1)	23 transcripts
S. lepidophylla	-	-	**2** (1/0)	**20** (9/9)	**13** (5/7)	**9** (1/1)	44 transcripts
S. denticulata	-	-	**3** (1/nd)	**16** (2/nd)	**9** (2/nd)	**9** (0/nd)	37 transcripts

The number of transcripts classified as MIPs in the *Selaginella* assemblies are indicated in **bold**. In brackets the proportion of these induced during DH/RH.

*MIPs encoded in the genome of *S. moellendorffii* identified by Anderberg *et al.* (2012).
