# Supplementary material for: Comparative transcriptome analysis suggests convergent evolution of desiccation tolerance in Selaginella species
Source: BMC Plant Biol. 2020 Oct 12;20:468. doi: 10.1186/s12870-020-02638-3 (PMC7549206; doi:10.1186/s12870-020-02638-3)
Supplement: Supplementary file 11 — Additional file 11: Table S3. Cell wall subcategories induced during DH and RH. [file 12870_2020_2638_MOESM11_ESM.pdf]

Table S3. Cell wall subcategories induced during DH and RH.

S. lepidophylla		
**Subcategory**	**DH**	**RH**
cell wall proteins.AGPs	3	3
cell wall proteins.RGP	2	2
cellulose synthesis	--	1
cellulose synthesis.cellulose synthase	1	1
cellulose synthesis.COBRA	3	1
degradation.cellulases and beta -1,4-glucanases	--	1
degradation.mannan-xylose-arabinose-fucose	--	1
degradation.pectate lyases and polygalacturonases	1	3
hemicellulose synthesis	3	1
hemicellulose synthesis.glucuronoxylan	1	1
modification	12	8
pectin*esterases.misc	1	--
pectin*esterases.PME	3	1
precursor synthesis.AXS	2	1
precursor synthesis.GMD	1	1
precursor synthesis.NDP sugar pyrophosphorylase	1	2
precursor synthesis.phosphomannomutase	1	1
precursor synthesis.UDP-Glc dehydrogenase (UGD)	3	4
precursor synthesis.UDP-glucose 4,6-dehydratase	1	1
precursor synthesis.UER	1	1
precursor synthesis.UGE	1	1
precursor synthesis.UXS	3	3
**TOTAL**	**44**	**39**

S. sellowii		
**Subcategory**	**DH**	**RH**
cell wall proteins	1	1
degradation.pectate lyases and polygalacturonases	7	3
hemicellulose synthesis	2	1
hemicellulose synthesis.glucuronoxylan	1	1
modification	1	--
pectin synthesis.homogalacturonan	1	--
pectin*esterases.PME	3	2
precursor synthesis.AXS	1	
precursor synthesis.GAE	1	1
precursor synthesis.KDO pathway	--	1
precursor synthesis.UER	1	1
precursor synthesis.UGE	1	--
precursor synthesis.UXS	1	--
**TOTAL**	**21**	**11**

S. denticulata		
**Subcategory**	**DH**	**RH**
cell wall proteins	1	--
cell wall proteins.AGPs	1	--
cell wall proteins.HRGP	1	--
cell wall proteins.RGP	1	--
cellulose synthesis	3	--
degradation.mannan-xylose-arabinose-fucose	3	--
degradation.pectate lyases and polygalacturonases	7	--
hemicellulose synthesis	4	--
hemicellulose synthesis.glucuronoxylan	1	--
modification	5	--
pectin*esterases.PME	1	--
precursor synthesis.sugar kinases	1	--
precursor synthesis.UXS	1	--
**TOTAL**	**30**	**--**
