# Supplementary material for: Comparative transcriptome analysis suggests convergent evolution of desiccation tolerance in Selaginella species
Source: BMC Plant Biol. 2020 Oct 12;20:468. doi: 10.1186/s12870-020-02638-3 (PMC7549206; doi:10.1186/s12870-020-02638-3)
Supplement: Supplementary file 12 — Additional file 12. Morphological identification, habitat description and coordinates. [file 12870_2020_2638_MOESM12_ESM.pdf]

Morphological identification, habitat description and coordinates.

Specimens identification according to morphological characteristics described in the "Key to the Mexican species of *Selaginella*" from Mickel and Smith (The Pteridophytes of Mexico, The New York Botanical Garden Press, 2004).

**Sample ID:**	42-18012017
**Scientific name:**	*Selaginella sellowii* Hieron.
**Location:**	San José del Chilar (locality), San Juan Bautista Cuicatlán, Oax, Mex.
	17° 42' 56.2" N, 96° 56' 28.39" W (711 m.a.s.l.)
**Vegetation:**	Tropical deciduous forest/scrub
**Date:**	18 January 2017

**Sample ID:**	50-27082017
**Scientific name:**	*Selaginella lepidophylla* (Hook. & Grev.) Spring
**Location:**	Tlacotepec (locality), Zacualpan de Amilpas, Mor, Mex.
	18° 49' 19.6" N, 98° 45' 10.29" W (1789 m.a.s.l.)
**Vegetation:**	Tropical open deciduous forest
**Date:**	27 August 2017
